# Plant Beneficial Deep-Sea Actinobacterium, *Dermacoccus abyssi* MT1.1^T^ Promote Growth of Tomato (*Solanum lycopersicum*) under Salinity Stress

**DOI:** 10.3390/biology11020191

**Published:** 2022-01-26

**Authors:** Pharada Rangseekaew, Adoración Barros-Rodríguez, Wasu Pathom-aree, Maximino Manzanera

**Affiliations:** 1 Doctor of Philosophy Program in Applied Microbiology (International Program) in Faculty of Science, Chiang Mai University, Chiang Mai 50200, Thailand; Pharada_ra@cmu.ac.th; 2 Graduate School, Chiang Mai University, Chiang Mai 50200, Thailand; 3 Department of Microbiology, Institute for Water Research, University of Granada, 18071 Granada, Spain; dorysbr@correo.ugr.es (A.B.-R.); manzanera@ugr.es (M.M.); 4 Research Center in Bioresources for Agriculture, Industry and Medicine, Department of Biology, Faculty of Science, Chiang Mai University, Chiang Mai 50200, Thailand

**Keywords:** *Dermacoccus abyssi*, marine actinobacteria, plant growth promoting actinobacteria, plant growth promoting actinomycetes, genomic analysis, salt tolerance, salinity stress, biosafety, sustainable agriculture, bioinoculants

## Abstract

**Simple Summary:**

Salt stress is an important environmental problem that negatively affects agricultural and food production in the world. Currently, the use of plant beneficial bacteria for plant growth promotion is attractive due to the demand for eco-friendly and sustainable agriculture. In this study, salt tolerant deep-sea actinobacterium, *Dermacoccus abyssi* MT1.1^T^ was investigated plant growth promotion and salt stress mitigation in tomato seedlings. In addition, *D. abyssi* MT1.1^T^ whole genome was analyzed for plant growth promoting traits and genes related to salt stress alleviation in plants. We also evaluated the biosafety of this strain on human health and organisms in the environment. Our results highlight that the inoculation of *D. abyssi* MT1.1^T^ could reduce the negative effects of salt stress in tomato seedlings by growth improvement, total soluble sugars accumulation and hydrogen peroxide reduction. Moreover, this strain could survive and colonize tomato roots. Biosafety testing and genome analysis of *D. abyssi* MT1.1^T^ showed no pathogenicity risk. In conclusion, we provide supporting evidence on the potential of *D. abyssi* MT1.1^T^ as a safe strain for use in plant growth promotion under salt stress.

**Abstract:**

Salt stress is a serious agricultural problem threatens plant growth and development resulted in productivity loss and global food security concerns. Salt tolerant plant growth promoting actinobacteria, especially deep-sea actinobacteria are an alternative strategy to mitigate deleterious effects of salt stress. In this study, we aimed to investigate the potential of deep-sea *Dermacoccus abyssi* MT1.1^T^ to mitigate salt stress in tomato seedlings and identified genes related to plant growth promotion and salt stress mitigation. *D. abyssi* MT1.1^T^ exhibited plant growth promoting traits namely indole-3-acetic acid (IAA) and siderophore production and phosphate solubilization under 0, 150, 300, and 450 mM NaCl in vitro. Inoculation of *D. abyssi* MT1.1^T^ improved tomato seedlings growth in terms of shoot length and dry weight compared with non-inoculated seedlings under 150 mM NaCl. In addition, increased total soluble sugar and total chlorophyll content and decreased hydrogen peroxide content were observed in tomato inoculated with *D. abyssi* MT1.1^T^. These results suggested that this strain mitigated salt stress in tomatoes via osmoregulation by accumulation of soluble sugars and H_2_O_2_ scavenging activity. Genome analysis data supported plant growth promoting and salt stress mitigation potential of *D. abyssi* MT1.1^T^. Survival and colonization of *D. abyssi* MT1.1^T^ were observed in roots of inoculated tomato seedlings. Biosafety testing on *D. abyssi* MT1.1^T^ and in silico analysis of its whole genome sequence revealed no evidence of its pathogenicity. Our results demonstrate the potential of deep-sea *D. abyssi* MT1.1^T^ to mitigate salt stress in tomato seedlings and as a candidate of eco-friendly bio-inoculants for sustainable agriculture.

## 1. Introduction

Agricultural productivity loss from salt stress is a serious issue that negatively impacts global food security [1,2]. Salinity can occur from natural causes, such as weathering of saline parent material and rise of saline groundwater, and anthropogenic activity, such as excessive use of underground water for irrigation and poor drainage [2,3]. It is expected that 30% of arable land loss will happen within the next 25 years and up to 50% within 2050 [4]. Salinity causes osmotic and oxidative stress which leads to a series of biochemical, molecular, morphological, and physiological changes that negatively affect plant growth and development [1,4,5,6]. In response to salt stress, plants apply various strategies to mitigate these negative effects. Plants can accumulate compatible solutes to balance osmotic pressure in the cells [7,8,9,10,11]. The most commonly observed osmoregulatory compounds are proline, soluble sugars (sucrose and trehalose), and glycine betaine [8,9,10,11,12]. In addition, the excessive generation of reactive oxygen species (ROS) from salt stress causes oxidative stress in plants which damage lipids, proteins, and nucleic acid in plant cells [5,11,13] and reduces photosynthesis [13]. Plants detoxify these excessive ROS by antioxidative enzymes, mainly catalase, peroxidase, and superoxide dismutase, and non-enzymatic antioxidants such as carotenoids and glutathione [9,10,11,12]. Although plants exhibit various mechanisms to survive in saline environments, these adaptative responses are species-dependent, which dictates the level of stress tolerance in each species [9]. Once the salinity level exceeds the maximum limit for growth, plants will not be able to survive.

There are several ways to mitigate salt stress in plants such as agronomic practices, the use of salt-tolerant crop species obtained either by genetic engineering or conventional breeding [3] and plant growth promoting bacteria [9]. Currently, the use of plant growth promoting bacteria is gaining more attention due to environmental awareness and sustainable food production concerns. Plant growth promoting bacteria, in particular actinobacteria, have been adopted to enhance crop production and alleviate salt stress in several plant species. Actinobacteria have been reported to promote plant growth under salt stress in tomato [14,15,16,17], soybean [18,19], wheat [20,21], alfalfa [22], and dwarf glasswort [23]. Recently, actinobacteria with plant growth promoting potential dwell in extreme saline environments such as marine sediments, mangrove, and halophyte are attracting research attention due to their adaptive features in such environments which are beneficial to plants under salinity stress. For example, *Nocardiopsis yanglingensis, Streptomyces jiujiangensis, S. psammoticus,* and *Pseudonocardia oroxyli* from a mangrove area in Thailand were able to promote the growth of rice seedlings under normal and NaCl up to 200 mM [24]; *Glutamicibacter halophytocola* KLBMP 5180 [16] and *Streptomyces* sp. KLBMP5084 [15] from root of halophyte *Limonium sinense* enhanced tomato seedling growth under salt stress condition; *Streptomyces* spp. and *Nocardiopsis* spp. from two saline sites in the northeast region of Algeria promoted the growth of wheat under 0.25 to 1 M NaCl [21]; and *D. barathri* MT2.1^T^ and *D. profundi* MT2.2^T^ from deep-sea sediments mitigated salt stress in tomato seedlings under 150 mM NaCl [17].

Actinobacteria promote plant growth and mitigate salt stress via various mechanisms either directly or indirectly. Direct mechanism involves the production of phytohormones such as indole-3-acetic acid, siderophore production and phosphate solubilization [25,26]. Growth improvement by IAA producing actinobacteria has been reported in *Arabidopsis* seedlings inoculated with *Streptomyces* PGPA39 in terms of plant biomass and number of lateral roots [14]. Furthermore, nutrient acquisition in plants can be improved by siderophore producing and phosphate solubilizing actinobacteria as exemplified in *Streptomyces* isolate C for wheat [20] and *Arthrobacter woluwensis* AK1 in soybean [18]. Actinobacteria can indirectly promote plant growth by means such as controlling phytopathogens by antibiotics and lytic enzymes and competition for nutrients and inducing systematic resistance (IST) [25,26].

In addition, actinobacteria mitigate salt stress or enhance salt tolerance ability in plants using several strategies: 1-aminocyclopropane-1-carboxylic acid (ACC) deaminase production, osmoregulation, and antioxidative defense/protection system. Osmoregulation can be achieved by the production of compatible solutes to relieve osmotic stress in plants under salt stress condition [27]. Common compatible solutes include amino acids (proline, glycine, and betaine) and sugars (sucrose and trehalose) [5,27,28,29]. Inoculation of *Streptomyces* sp. KLBMP 5084 in tomato plants increased soluble sugar content in leaf and stem and proline content in stem under 200 mM NaCl [15]. *Glutamicibacter halophytocola* KLBMP 5180 increased proline content in leaves and stem of tomato seedlings with and without salt stress [16].

Oxidative stress as a result of salinity enhances the production of reactive oxygen species (ROS) such as hydrogen peroxide. There are reports that actinobacteria are able to regulate ROS levels in plants under stress through enzymatic (catalase, peroxidase, and superoxide dismutase) and non-enzymatic antioxidative systems [30,31]. For instance, halotolerant *G. halophytocola* KLBMP 5180 promoted the growth of tomato seedlings and mitigated salt stress by several mechanisms: increased proline content, antioxidant enzymes production (catalase and peroxidase), and ion homeostasis [16]. Inoculation of *Streptomyces* sp. KLBMP5084 increased catalase and peroxidase production in tomato seedlings under salt stress [15].

Higher ethylene levels in plants under abiotic stress, including salt stress, causes several damage to plants, for example leaf yellowing, plant organs senescence, the abscission of leaves, petals, and flowers, and premature death [32]. Actinobacteria can alleviate salt stress by degrading ACC, an ethylene precursor using the enzyme ACC deaminase [31,32] and promote the growth of crop plants such as pepper [28] and tomato [14].

As the use of microorganisms to promote plant growth in agricultural production is gaining popularity, there is a concern about the safety of plant growth promoting microorganisms on biodiversity in environment and health of human, animals and plants. The European Parliament released the regulation (EU) 2019/1009 on biostimulants to protect humans, plants, animals, and the environment [33]. To comply with current regulations, it is necessary to consider including experiments to guarantee the safety of the strains under study.

The type strain of *D. abyssi* MT1.1^T^ was previously isolated from the Challenger Deep sediments of the Mariana Trench by our group and identified as a piezotolerant strain [34]. We opine that, with its ability to survive under constant salinity and high pressure, *D. abyssi* MT1.1^T^ would be able to mitigate adverse effects of salinity stress on plant growth. Recently, the whole genome sequence of *D. abyssi* MT1.1^T^ has been released [35]. However, the relationship between genes responsible for and the plant beneficial traits in *D. abyssi* MT1.1^T^ has not been explored. Therefore, we aim to investigate the potential of *D. abyssi* MT1.1^T^ to promote the growth of tomato seedlings under salt stress. In addition, its whole genome was analyzed for genes related to plant growth promotion in order to understand salt stress mitigation mechanisms at molecular level. We also evaluated the safety of *D. abyssi* MT1.1^T^ on viability of *Escherichia coli* MC4100 and soil nematodes (*Caenorhabditis elegans*).

## 2. Materials and Methods

All methods used are summarized in a diagram as shown in Figure S1.

### 2.1. Bacterial Strains and Growth Conditions

The type strain of *Dermacoccus abyssi* MT1.1^T^ used in this study was previously isolated from the Mariana Trench sediment in northwest Pacific Ocean [36]. For the biosafety test, *Escherichia coli* OP50, *E. coli* MC4100, *Pseudomonas putida* KT2440, and *P. aeruginosa* PA14 were used. The bacteria were routinely grown and maintained on International *Streptomyces* Project 2 (ISP2) agar or Tryptic Soy agar (TSA) as a working stock. All bacteria are also maintained as cell suspension in 20% glycerol at −20 °C for long term preservation.

### 2.2. Assessment of Plant Growth Promoting Traits of Actinobacteria

Three plant growth promoting properties (IAA, siderophore production, and phosphate solubilization) of *D. abyssi* MT1.1^T^ were determined under induced salinity stress at 150, 300, and 450 mM NaCl and without salinity. *D. abyssi* MT1.1^T^ was cultured in glucose yeast extract (GYE) broth amended with 2 mg mL^−1^ L—tryptophan on a shaker at 110 rpm for 7 days in the dark under different NaCl concentrations. Supernatant was collected and indole-3-acetic acid (IAA) production was determined using standard colorimetric method as described by Lasudee et al. [37].

Siderophore production was detected on Chrome-azurol S (CAS) agar [38], incubated at 28 °C for 7 days in the dark. Siderophores type were determined and quantified in King’s B broth supplemented with different NaCl concentrations, incubated at 28 °C for 7 days, using standard methods as described by Rangseekaew et al. [17].

Solubilization of inorganic phosphate was evaluated on Pikovskaya’s agar (PVK) containing 0.5% (*w*/*v*) tri-calcium phosphate and incubated at 28 °C for 7 days. Quantification of released phosphorous was carried out in PVK broth containing 0.5% (*w*/*v*) tri-calcium phosphate using colorimetric method as described previously [17].

ACC deaminase activity was assessed on DF minimal salt medium supplemented with three different nitrogen sources: 3 mmol L^−1^ ACC, 3 mmol L^−1^ (NH_4_)_2_SO_4_ (positive control), and no nitrogen source (negative control) followed the procedure described by Palaniyandi et al. [14].

Catalase test was determined according to Djebaili et al. [39]. One loop full of *D. abyssi* MT1.1^T^, grown on GYE agar at room temperature for 7 days, was added to 3% hydrogen peroxide solution on a glass slide. The rapid bubble that occurs indicated the ability to produce catalase enzyme.

Ammonia production was evaluated according to Djebaili et al. [39]. One loop full of *D. abyssi* MT1.1^T^ was cultured in peptone broth incubated at room temperature for 7 days on a shaker at 110 rpm. A change of color from faint yellow to dark brown after the addition of Nessler’s reagent indicated ammonia production.

### 2.3. Genomic Analysis for Plant Growth-Promoting Properties

Whole genome sequence of *D. abyssi* MT1.1^T^ was previously published [35] and is available in DDBJ/EMBL/GenBank databases under the accession number QWLM00000000. In this study, some gene encoding for proteins that are recognized as important for plant growth promotion and mitigation mechanisms related to salinity stress were identified in the genome using the Rapid Annotation Subsystem Technology server (RAST) server tools version 2.0 [40], Anti-SMASH version 6.0 [41], and PRISM3 [42]. The pathogenicity prediction of *D. abyssi* MT1.1^T^ was analyzed using PathogenFinder 1.1 web-server [43].

### 2.4. Promotion of Tomato Growth by D. abyssi MT1.1^T^ under Salt Stress Condition

#### 2.4.1. Plant Growth Condition and Bacterial Inoculation

Tomato (*Solanum lycopersicum*, Raf tomato) was chosen as a model plant to study the potential of *D. abyssi* MT1.1^T^ to mitigate salt stress. *D. abyssi* MT1.1^T^ was previously tested to have no negative effect in plants without stress (data not shown). One-month-old tomato seedlings (SaliPlant S.L. specialist grower, Granada, Spain) were inoculated with *D. abyssi* MT1.1^T^ cells at 10^8^–10^9^ CFU/mL in 0.5 M9 buffer [44]. All tomato plants were subjected to salinity stress at 150 mM NaCl. Non-inoculated tomato seedlings with and without salinity stress were used as control. This time was considered as day 0 for salinity stress. Tomato plants were maintained in a growth room at constant relative humidity (50–60%), a photoperiod of 12 h day/night cycle, the temperature was changed from 18 to 20 °C for the night cycle to 20–25 °C in the diurnal [45]. After 14 days of inoculation, fresh weight (FW), dry weight (DW), root length, and shoot length were recorded. Relative water content (RWC) was determined by the following equation {RWC (%) = [(FW − DW)/(TW − DW) × 100]} as described by Oukarroum et al. [46]. Membrane stability index (MSI) was calculated as MSI = [1 − (C1/C2)] × 100 according to Asharf et al. [47]. Biochemical properties of tomato seedlings were also determined: chlorophyll contents were determined according to Arnon [48]. Total chlorophyll content was calculated from the following equations: total chlorophyll (mg g^−1^) = 20.2 × OD_645_ + 18.2 × OD_663_ × V/(1000 × W). Proline content in plants was assayed using colorimetric method according to Bates et al. [49]. Total soluble sugar content (TSS) was estimated as described by Dubois et al. [50]. Hydrogen peroxide (H_2_O_2_) concentration was examined following the method of Velikova et al. [51].

#### 2.4.2. Root Colonization by *D. abyssi* MT1.1^T^

Tomato seeds were surface-sterilized according to Botta et al. [52]. Briefly, seeds were wrapped in a cloth sheet and treated with 70% ethanol (*v*/*v*) for 3 min, followed by 5% NaClO for 5 min with constant mixing. Seeds were subsequently washed 6 times in sterile distilled water. Seeds were inoculated by immersion in *D. abyssi* MT1.1^T^ cell suspension (10^−7–^10^−8^ CFU mL^−1^) for 3 h on a shaker at 120 rpm. Control seeds were prepared with the same procedure using sterile distilled water and air-dried for 3 min under aseptic conditions. Inoculated and non-inoculated seeds were cultivated in a glass bottle containing 50 mL of half-strength Murashige and Skoog (MS) media supplemented with 3% sucrose and 0.8% agar (modified from Djebaili et al. [39]) and incubated in a growth chamber with controlled environment (25 °C, photoperiod 16 h light and 8 h dark, 65% relative humidity, active photo-synthetical radiation 120 µmol m^−2^s^−1^). After 25 days, tomato roots were taken to determine the colonization ability of *D. abyssi* MT1.1^T^ as follows: One gram of non-sterile root was grounded in 9 mL of 0.85% NaCl solution and the resultant root suspension was 10-fold serially diluted and spread on GYE agar plates supplemented with 25 µg mL^−1^ nalidixic acid and 25 µg mL^−1^ nystatin and incubated at room temperature for 72 h. Colony of *D. abyssi* MT1.1^T^ were counted and expressed as CFU/g root fresh weight. Putative *D. abyssi* MT1.1^T^ was confirmed by 16S rRNA gene sequencing. Dried-root samples were coated with gold and observed using LV-Scanning Electron Microscope JSM 5910 using the service of Electron Microscope Research and Service Center, Faculty of Science, Chiang Mai University, Thailand.

### 2.5. Biosafety Test for Actinobacteria

#### 2.5.1. Pathogenicity Bioassay Based on *Caenorhabditis elegans*

This bioassay was carried out as described by Vílchez et al. [53]. *D. abyssi* MT1.1^T^ culture (100 µL) was dropped as T-shaped line on five potatoes dextrose agar (PDA) plates and kept at 30 °C for 3 h to dry zone of inoculation. Plates were maintained at 22 °C for 24 h before use. Five juvenile nematodes (larval stage L3–L4) were added to each plate, incubated at 22 °C, and the number of adults, juveniles, deposited eggs, and dead nematodes were counted under microscope at 20× and 40× magnification every 24 h for 7 days. *Pseudomonas aeruginosa* PA14 was used as a pathogenic control and *Escherichia coli* OP50 as a non-pathogenic control for natural growth and death rate estimation of nematode.

#### 2.5.2. *Escherichia coli* MC4100 Sensitivity

This bioassay was carried out followed the method described by Vílchez et al. [53]. Five milliliters of *E. coli* MC4100 (10^8^–10^9^ CFU/mL) in M9 buffer were mixed with 1 mL filtered sterile supernatant of *D. abyssi* MT1.1^T^. The mixture was incubated at room temperature for 1 h, then serially diluted and plated on tryptic soy agar to estimate the number of the remaining *E. coli* MC4100 (CFU/mL). *E. coli* suspension mixed with *Pseudomonas putida* KT2440 supernatant was used as a non-pathogenic control and with *P. aeruginosa* PA14 as a pathogenic control [54,55].

### 2.6. Statistical Analysis

The data from in vitro and in vivo plant growth promoting experiments were repeated three times and presented as mean values and standard deviation (SD). All data were analyzed with one way analysis of variance (ANOVA) for Completely Randomized Designs (CRD) and Duncan’s multiple range tests. Calculation was carried out using SPSS (version 17.0) at *p* < 0.05. Significantly different mean values were indicated with different letters.

## 3. Results

### 3.1. Assessment of Plant Growth Promoting Traits of Actinobacteria

Deep-sea *D. abyssi* MT1.1^T^ showed the ability to produce IAA in GYE broth supplemented with tryptophan with and without NaCl (Table 1). The highest concentration of IAA of 37.50 µg mL^−1^ was detected in culture broth without NaCl. Under salinity at 150, 300, and 450 mM NaCl, the IAA production was decreased to 8.36–11.75 µg mL^−1^. However, there is no significant difference in the quantity of IAA production under each NaCl concentration. The production of siderophores by *D. abyssi* MT1.1^T^ was observed on Chrome-azurol S (CAS) plate assay as a yellow zone around the agar plug. Quantitative analysis in culture broth showed that hydroxamate type was produced significantly higher than catecholate type siderophores at 46.67 µmol mL^−1^ and 2.98 µmol mL^−1^, respectively. Under salt stress (150, 300, and 450 mM NaCl), higher quantities of hydroxamate siderophores was produced as compared to without NaCl (170.83–189.17 µmol mL^−1^). *D. abyssi* MT1.1^T^ increased the production of catecholate type under 150 mM NaCl; however, the production was decreased at 300mM and 450 mM NaCl. *D. abyssi* MT1.1^T^ produced clear zone of phosphate solubilization on PVK agar and released 71.62 µg mL^−1^ of phosphorus in culture broth. At higher NaCl concentrations (150 and 300 mM), released phosphorus showed no significant difference compared with no NaCl. At 450 mM NaCl, the lowest released phosphorus was measured (62.33 µg mL^−1^). A similar pH change of culture broth was observed with and without NaCl (7 to 5.1). For ACC deaminase activity, *D. abyssi* MT1.1^T^ grew poorly on DF minimal salt medium compared to minimal medium supplemented with ammonium sulphate (positive control). Catalase activity was positive for *D. abyssi* MT1.1^T^ to break down hydrogen peroxide (H_2_O_2_) to water and oxygen. For ammonification test, the color of culture broth was changed to dark yellow after adding Nessler’s reagent. Hence, *D. abyssi* MT1.1^T^ could convert amino acids and produce ammonia.

### 3.2. Genomic Analysis for Plant Growth-Promoting Properties and Stress Response

A whole genome sequence of *D. abyssi* MT1.1^T^ was annotated using RAST server; the estimated genome size was approximately 3.16 Mb with the GC content of 68.1% [35]. Gene annotation in RAST server predicted genes into 27 subsystems (Figure S2.) with 3091 predicted coding sequences. The major subsystems are composed of gene encoding for amino acids and derivatives (19.9%), protein metabolism (14.8%), and carbohydrates (12.7%). Plant growth promoting related genes were summarized in Table 2. Ten genes involved in tryptophan biosynthesis were detected in amino acids and derivatives subsystem, in which tryptophan is the main substrate for indole-3 acetic acid production (IAA). *D. abyssi* MT1.1^T^ contained several genes responsible for plant nutrient acquisition including iron, phosphorus, and nitrogen. For iron, a gene predicted for siderophore producing enzyme, NRPS-independent siderophore synthase was identified by PRISM3 algorithm. Using RAST server, three protein encoding genes related to ferrous iron transporter EfeUOB were found which functioned to directly import ferrous iron at low pH. Gene encoding for phosphate metabolizing enzymes, alkaline phosphatase; inorganic pyrophosphatase (catalyze the hydrolysis of inorganic pyrophosphate PPi to inorganic phosphate Pi) [56]; and poly-phosphorus hydrolyzing enzymes such as polyphosphate kinase, polyphosphate glucokinase, and exopolyphosphatase were observed. In addition, other genes related to the uptake and transport of inorganic phosphate in PHO regulon were presented. Eight genes associated with bacterial ammonium assimilation were also detected.

Three subsystem genes involved in osmotic stress response were found (Table 2). The predicted gene for glycerol uptake facilitator protein, which functions in osmoregulation, was found. Genes responsible for ectoine biosynthesis and regulation, choline and betaine uptake, and betaine biosynthesis were also found. The ectoine biosynthetic gene cluster was also detected by Anti-SMASH version 6.0. In addition, two genes, glutathionylspermidine synthase and alkyl hydroperoxide reductase subunit C-like protein related to oxidative stress were detected by RAST.

Analysis of *D. abyssi* MT1.1^T^ genome using PathogenFinder 1.1 web server showed no protein related to protein families associated with pathogenic strains. Gene prediction for superoxide dismutase was also presented in its genome which shared high identity (87.22%) with superoxide dismutase in non-pathogenic actinobacterium, *Nocardioides* sp. JS614.

### 3.3. Promotion of Tomato Growth by D. abyssi MT1.1^T^ under Salt Stress Condition

**Figure 1:**
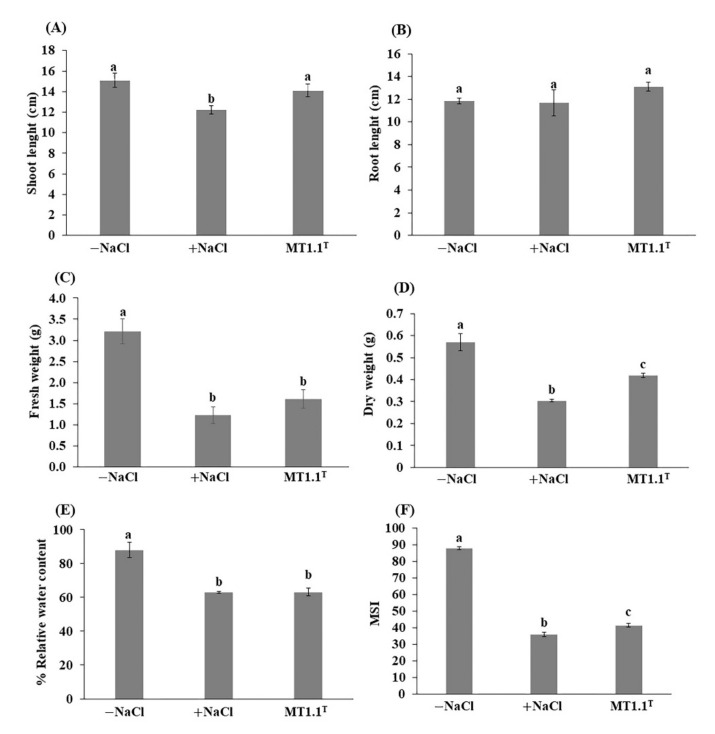
Promotion of tomato seedlings growth by *D. abyssi* MT1.1^T^ under 150 mM NaCl. (**A**) shoot length, (**B**) root length, (**C**) fresh weight, (**D**) dry weight, (**E**) % relative water content, and (**F**) membrane stability index (MSI). Data represent the mean values of three replicates. Different letters (a, b, and c) indicate a significant difference according to Duncan at *p* < 0.05.

**Figure 2:**
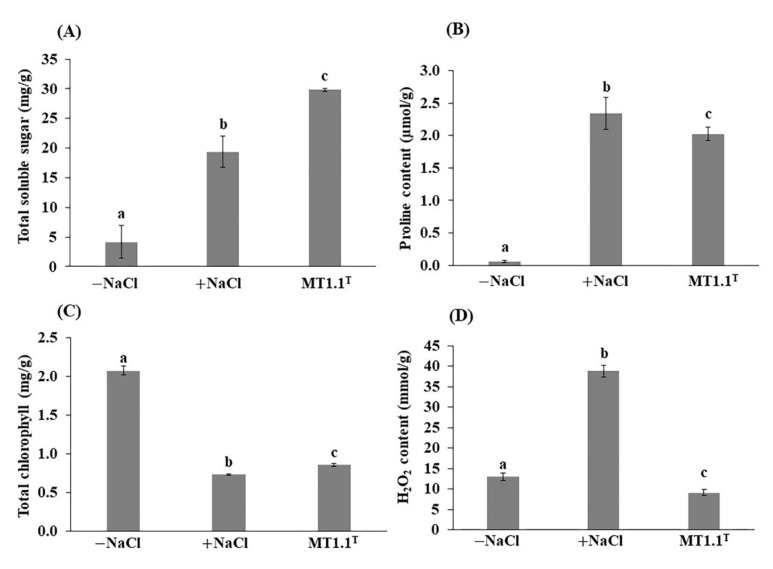
Effect of *D. abyssi* MT1.1^T^ on biochemical parameters of tomato seedlings under 150 mM NaCl. (**A**) total soluble sugar, (**B**) proline content, (**C**) total chlorophyll content, and (**D**) hydrogen peroxide content. Data represent the mean values of three replicates. Different letters (a, b, and c) indicate a significant difference according to Duncan at *p* < 0.05.

The promotion of tomato growth by deep-sea *D. abyssi* MT1.1^T^ under 150 mM NaCl are shown in Figure 1 and Figure 2. At the end of experiment, tomato inoculated with *D. abyssi* MT1.1^T^ showed shoot length (Figure 1A) and dry weight (Figure 1D) significantly higher than non-inoculated tomato with salt stress. Inoculation of this strain showed fresh and dry weight significantly lower than non-inoculated tomato without stress (Figure 1C,D). For physiological parameters, relative water content (% RWC) value of *D. abyssi* MT1.1^T^ inoculated tomato was not significantly different compared to non-inoculated tomato with salt stress (Figure 1E). The membrane stability index of tomato inoculated with *D. abyssi* MT1.1^T^ was significantly higher than non-inoculated tomato under salt stress (Figure 1F).

Biochemical changes in tomato after inoculation with *D. abyssi* MT1.1^T^ under salt stress were analyzed in terms of total soluble sugar, proline content, total chlorophyll content, and hydrogen peroxide (H_2_O_2_) content (Figure 2A–D). For total soluble sugar (TSS), the highest TSS was recorded in tomato inoculated with *D. abyssi* MT1.1^T^ compared to non-inoculated with and without salt stress (Figure 2A). The proline content of *D. abyssi* MT1.1^T^ inoculated tomato showed lower than non-inoculated tomato under salt stress, but significantly higher than non-inoculated tomato without salt stress (Figure 2B). For total chlorophyll content, *D. abyssi* MT1.1^T^ increased total chlorophyll content in inoculated tomato compared to non-inoculated tomato under salt stress (Figure 2C). However, chlorophyll content of non-inoculated and inoculated tomato under salt stress were significantly lower than non-inoculated tomato without salt stress. Hydrogen peroxide content in tomato was analyzed and showed that the lowest H_2_O_2_ was found in *D. abyssi* MT1.1^T^ inoculated tomato, followed by non-inoculated tomato without stress and non-inoculated tomato with stress (Figure 2D).

#### Tomato Root Colonization by *D. abyssi* MT1.1^T^

**Figure 3:**
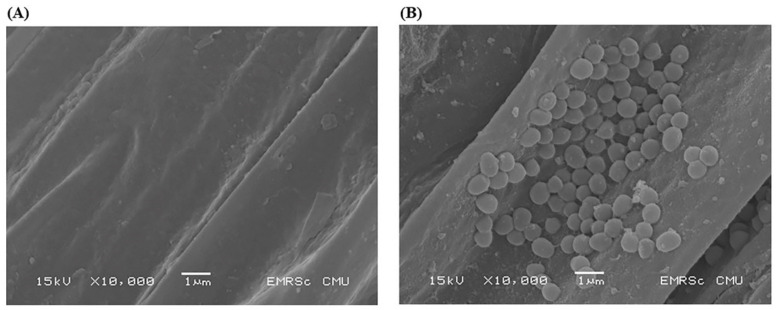
Scanning electron micrographs of tomato roots obtained from 25-day-old tomato seedlings. (**A**) non-inoculated tomato seedlings, (**B**) *D. abyssi* MT1.1^T^-inoculated tomato seedlings.

The total of 3.87 × 10^7^ CFU/g *D. abyssi* MT1.1^T^ were re-isolated from inoculated-tomato roots. No microbial growth was observed from non-inoculated roots. The confirmation of putative *D. abyssi* MT1.1^T^ by 16S rRNA gene sequencing showed 98.7% similarity with *D. abyssi* MT1.1^T^. The colonization of *D. abyssi* MT1.1^T^ in tomato roots was confirmed by SEM analysis. SEM micrograph showed distribution of a characteristic cluster of circular cells of *D. abyssi* MT1.1^T^ on the root surface (Figure 3B). The control roots revealed no sign of microbial colonization (Figure 3A).

### 3.4. Biosafety Test for Actinobacteria 

#### 3.4.1. Pathogenicity Bioassay Based on *Caenorhabditis elegans*

**Figure 4:**
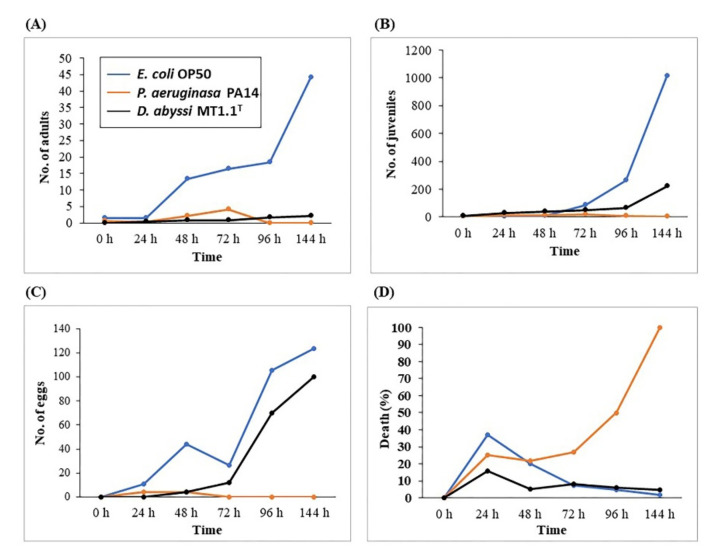
The pathogenicity assay of deep-sea *D. abyssi* MT1.1^T^ on the survival of *C. elegans.* Time course of changes in the number of (**A**) adults, (**B**) juveniles, (**C**) eggs, and (**D**) death. Data represent the mean values of three replicates.

The pathogenicity of deep-sea *D. abyssi* MT1.1^T^ on the survival of *C. elegans* was carried out based on the number of adults, juveniles, eggs, and dead nematodes (Figure 4A–D). The lowest numbers of adults, juveniles, eggs were found in nematodes fed with *P. aeruginosa* PA14 (as pathogenic strain) and all nematodes died after 96 h. The highest numbers of adults, juveniles, and eggs were found in nematodes fed with *E. coli* OP50 (as non-pathogenic strain). Nematodes fed with *D. abyssi* MT1.1^T^ showed a decrease in numbers of adults (95%) and juveniles (78%) compared to control *E. coli* OP50. At 144 h, numbers of eggs from nematodes fed with *E. coli* OP50 and *D. abyssi* MT1.1^T^ were recorded at 124 eggs and 100 eggs, respectively. The percentage of deaths at 144 h, *P. aeruginosa* PA14 showed the highest death (100%), followed by *D. abyssi* MT1.1^T^ (5%), and *E. coli* OP50 (1.6%).

#### 3.4.2. *Escherichia coli* MC4100 Sensitivity

**Figure 5:**
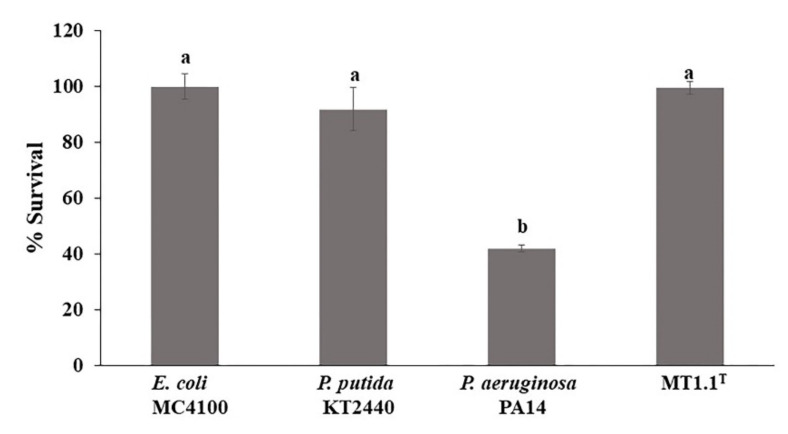
*Escherichia coli* MC4100 sensitivity. Different letters (a,b) indicate a significant difference according to Duncan at *p* < 0.05.

The effect of secondary metabolites released by *D. abyssi* MT1.1^T^ on the microbial community was studied using *E. coli* MC4100 cells (Figure 5). The percentage of survival in *E. coli* MC4100 cells exposed to cell free supernatants of *D. abyssi* MT1.1^T^ (99%), *E. coli* MC4100 (100%), and *P. putida* KT2440 (non-pathogenic strain) (92%) were similar (*p* < 0.05). However, only 42% of *E. coli* MC4100 cells survived after exposed to cell free supernatants of *P. aeruginosa* PA14 (pathogenic strain) for 1 h.

## 4. Discussion

### 4.1. Assessment of Plant Growth Promoting Abilities of Actinobacteria

Actinobacteria are well-known plant growth promoting bacteria which promote plant growth using various mechanisms both directly and indirectly [57]. In this research, we investigated three plant growth promoting abilities (IAA and siderophore production and phosphate solubilization) of deep-sea *D. abyssi* MT1.1^T^. Indole-3-acetic acid (IAA) is a major form of phytohormone auxin [58], which plays an important role in plant growth and development such as seed germination, embryo and fruits development, and root formation [26,57]. *D. abyssi* MT1.1^T^ produced IAA both with and without salt stress. A reduction in IAA production could be expected in bacteria under increasing NaCl concentration, however the reduction may not be related to the level of salt stress. In this study, IAA production by *D. abyssi* MT1.1^T^ at 150 mM NaCl was three-fold decreased as compared to those production at 0 mM NaCl. Similarly, the reduction in IAA production by *D. profundi* MT2.2^T^ (decreased from 12.20 to 7.73 µg mL^−1^) and *D. nishinomiyaensis* DSM20448^T^ (decreased from 16.64 to 9.39 µg mL^−1^), was recorded at 150 mM NaCl [17]. There is some evidence that IAA production is increased with increasing NaCl concentration. Sadeghi et al. [20] also reported the incremental increase in IAA production by *Streptomyces* isolating C in the presence of salt at 100–300 mM NaCl. However, IAA production does not necessarily decrease under salt stress [17]. For example, *D. profundi* MT2.2^T^ and *D. nishinomiyaensis* DSM20448^T^ produced higher IAA when added NaCl at 300 and 450 mM [17]. IAA production was not affected by NaCl addition as in the case of *D. barathri* MT2.1^T^ (150–450 mM NaCl).

Siderophores are specific iron chelating substance products from plants and microorganisms including actinobacteria under iron limitation. They play an important role in environmental iron (Fe^3+^) binding and make it available for microorganisms and plants [59]. Our results showed the ability of *D. abyssi* MT1.1^T^ to produce siderophores, particularly under NaCl condition (150–450 mM). The quantity of hydroxamate siderophore in culture broth with NaCl was three times higher than without NaCl condition. The highest catecholate siderophore production was observed at 150 mM NaCl. The production of catecholate siderophore was negatively influenced by higher NaCl concentration as fifty percent reduction was observed at 300–450 mM NaCl. On the contrary, siderophore producing zone of *Streptomyces* isolate C was enlarged in the presence of 100–300 mM NaCl on CAS agar plate assay [20]. From our results and previous study, NaCl concentration might affect siderophore production in a taxa specific manner. However, the exact influential mechanisms of NaCl on siderophore production require further investigation.

Phosphorus is the second essential macronutrient for plant growth. Although plants can absorb phosphorus in soil, only 0.1% of soil phosphorus is available for plants [60,61]. A large portion of phosphorus is in insoluble inorganic or organic forms. Various microorganisms possess phosphate solubilizing potential including actinobacteria [62]. In this present study, *D. abyssi* MT1.1^T^ showed phosphate solubilization ability with and without salt stress. The amount of phosphorus released in culture broth by *D. abyssi* MT1.1^T^ was slightly decreased with an increasing NaCl concentration. Similarly, salt concentration (100–300 mM) reduced the phosphate solubilization ability of *Streptomyces* isolate C [20]. It is evident that the presence of NaCl has negative effect on phosphate solubilization. It is well accepted that the main mechanisms of phosphate solubilization is the production of organic acids. The hydroxyl and carboxyl groups of acids can chelate cations such as calcium from insoluble phosphates and release phosphorus to the surrounding environment [61,62]. This phenomenon is easily observed by the decreasing of pH in the culture broth as in the case of this study and in the previous literature [17,20,37].

1-aminocyclopropane-1-carboxylate (ACC) is a precursor of ethylene, a plant stress hormone which is generated under stress condition and negatively affected on plant growth [63]. ACC deaminases producing bacteria, including actinobacteria, and could reduce ethylene levels by the production of ACC deaminase enzyme, which is responsible for the degradation of ACC to ammonia and α-ketobutyrate. However, deep-sea *D. abyssi* MT1.1^T^ showed no ACC deaminase activity. Therefore, it is possible that this deep-sea strain may use other mechanisms to reduce stress in plants.

### 4.2. Genomic Analysis for Plant Growth-Promoting Properties

Genomic analysis confirmed the presence of genes encoding for plant growth promoting properties in *D. abyssi* MT1.1^T^. For IAA production, genes responsible for tryptophan synthetic pathway were detected. Tryptophan is a main precursor of IAA biosynthetic pathway. There are two mechanisms of microorganisms for IAA biosynthesis: tryptophan-dependent and tryptophan-independent pathways [64]. Whole genome sequence of *D. abyssi* MT1.1^T^ contained tryptophan biosynthesis enzymes encoding genes including indole-3-glycerol phosphate synthase, tryptophan synthase alpha chain, tryptophan synthase beta chain, anthranilate synthase, and acting phosphoribosyl anthranilate isomerase. Indeed, *D. abyssi* MT1.1^T^ produced IAA in culture broth, supplemented with L-tryptophan (Table 1). These observations indicated that this strain synthesizes IAA via tryptophan-dependent pathway. Similarly, genes encoding for indole-3-glycerol phosphate synthase, tryptophan synthase alpha chain, and tryptophan synthase beta chain were reported from another plant growth promoting actinobacterium, *Micromonaspora chalcia* CMU55-4. This strain produced 11.35 µg mL^−1^ of IAA in culture media supplemented with L-tryptophan [65]. Recently, gene encoding for indole-3-glycerol phosphate synthase relating to IAA production was also detected in *Streptomyces adelaidensis* CAP261^T^ [66]. In addition, genes involving in three tryptophan related IAA biosynthetic pathways, indole 3-pyruvate, indole 3-acetamide, and tryptamine were detected in plant growth promoting *Streptomyces* strains from herbal vermicompost [67]. Moreover, *D. abyssi* MT1.1^T^ was previously shown to produce IAA in culture broth without L-tryptophan (data not shown). Genome analysis using RAST server also revealed gene predicted for indole-3-glycerol phosphate synthase. This enzyme is responsible for the formation of indole-ring, an essential step in IAA biosynthetic pathway [68]. Our results suggested that *D. abyssi* MT1.1^T^ is also capable of IAA production via tryptophan-independent pathway. Similarly, IAA production without L-tryptophan supplement was observed in *M. chalcia* CMU55-4 [65]. Its genome also contains indole-3-glycerol phosphate synthase. In addition, other actinobacteria were reported to produce IAA without L-tryptophan, for example members of the genera *Rhodococcus* [69] and *Streptomyces* [37,69,70].

Iron is the essential element for bacterial growth. However, in the ocean surface waters, only 0.01–2 nM of iron is available. The requirement of iron for bacterial growth is around 0.1 µM–1 mM [71,72]. Bacteria can employ ferric ion chelators in particular siderophores for ferric iron uptake [73]. *D. abyssi* MT1.1^T^ could produce hydroxamate and catecholate type siderophores in vitro. These siderophores are capable of chelating ferric iron, which provide iron for growth of *D. abyssi* MT1.1^T^. Although no siderophore gene homolog could be identified, genome analysis using RAST server predicted iron transport peroxidase EfeB, ferrous iron transport permease EfeU, and ferrous iron transport periplasmic protein EfeO, which are responsible for ferrous iron uptake under iron limitation conditions. Ferrous iron is predominant under low oxygen conditions such as marine sediment. As oxygen solubility is generally reduced with an increasing depth in marine environments, it is not surprising that ferrous iron transporters were found in the genome of deep-sea *D. abyssi* MT1.1^T^. The presence of genes responsible for iron transport and uptake suggested iron sequestration ability in *D. abyssi* MT1.1^T^. Previously, *D. barathri* MT2.1^T^ and *D. profundi* MT2.2^T^ were reported to produce both hydroxamate and catecholate type siderophores [17]. Similarly, genes related to the production of both siderophores could not be detected by RAST (data not shown). However, NRPS-independent siderophore synthase was identified by PRISM3 algorithm. From these findings, we hypothesized that deep-sea *Dermacoccus* species may produce iron chelating compound with unknown structure useful in biotechnological applications which warrants further investigation.

*D. abyssi* MT1.1^T^ showed the ability to solubilize tri-calcium phosphate in culture broth and pH of the culture broth was dropped from 7 to 5.1, indicating this strain produced organic acids for phosphate solubilization. Phosphate solubilization via organic acid production is common in several members of actinobacteria such as *Streptomyces* [37], including marine actinobacteria, *Kocuria palustris* [74], *D. barathri* MT2.1^T^, and *D. profundi* MT2.2^T^ [17]. Again, genes responsible for acid production were not predicted in *D. abyssi* MT1.1^T^ genome using RAST server. However, genes involved in phosphate metabolism were identified. Phosphate-regulated genes for high-affinity uptake of phosphate were observed, including phosphate regulon transcriptional regulatory protein PhoB (SphR), phosphate transport system regulatory protein PhoU, and phosphate regulon sensor protein PhoR (SphS). Under Pi limitation, Pho B acts as a kinase, which is activated by Pho R. On the contrary, under abundant Pi, PhoB acts as phosphatase which is interrupted by PhoR [75]. Furthermore, a low-affinity inorganic phosphate transporter encoding gene was found. It prefers Zn^2+^ or Mg^2+^, forming a neutral metal-phosphate complex (Me-PO_4_) [76]. Genes related to alkaline phosphatase were also found in the *D. abyssi* MT1.1^T^ genome. Alkaline phosphatase plays an important role in the utilization of phosphoesters, one of the most abundant groups of dissolved organic phosphorus in the ocean [77,78]. In addition, a gene encoded for exopolyphosphatase, an enzyme for the hydrolysis of inorganic polyphosphate, was found. Moreover, the genome of *D. abyssi* MT1.1^T^ showed protein kinase encoding genes. Protein kinases are enzymes that add phosphate group (PO_4_^3−)^ to other molecules (phosphorylation) such as amino acids, nucleic acids, and lipids, for example polyphosphate glucokinase, polyphosphate kinase, and polyphosphate kinase 2.

Nitrogen is necessary for bacterial cell growth such as proteins, nucleic acids, and cell wall components [79]. *D. abyssi* MT1.1^T^ mineralized amine or amide groups in peptone and converted them to ammonia/ammonium via ammonification process. Bacteria can assimilate ammonium, an inorganic nitrogen compound, to produce organic macromolecules for various cellular functions. In the present study, genome of *D. abyssi* MT1.1^T^ contained genes involved in ammonium assimilation including ferredoxin-dependent glutamate synthase, glutamine synthetase type I, glutamate synthase [NADPH] large chain, and glutamate synthase [NADPH] small chain. At low ammonia concentration (below 1 mM), high affinity-glutamine synthetase and glutamate synthase play an important role in ammonium assimilation [79]. In addition, a gene encoded for ammonium transport, which is responsible for ammonium uptake under limited ammonium, was identified. These findings provide evidence confirming the in silico prediction by RAST server. It is interesting to note that genes encoded for proline and trehalose production, which is responsible for mitigation mechanisms in plants under stress, were also detected and will be discussed in the next section.

### 4.3. Promotion of Tomato Growth by D. abyssi MT1.1^T^ under Salt Stress Condition

Salinity is a major constraint that threatens plant growth and productivity. Interestingly, the negative effect of salt stress could be mitigated by plant growth promoting actinobacteria with salt tolerant properties. In the present study, salinity at 150 mM NaCl negatively affected the growth of tomato seedlings as clearly seen by the reduction in shoot length (Figure 1A), fresh weight (Figure 1C), and dry weight (Figure 1D). Inoculation of tomato seedlings by *D. abyssi* MT1.1^T^ increased shoot length and dry weight compared to non-inoculated tomato under salt stress. These results are consistent with previous findings that the inoculation with deep-sea *D. barathri* MT2.1^T^ and *D. profundi* MT2.2^T^ showed better growth parameters under salt stress [17]. Two important physiological parameters that define salt stressed status of plants are RWC and MSI value [47]. Salt stress induces high salt accumulation in soil around the plant root. This accumulation increases soil osmotic potential, which limits plant water uptake [5] and lower RWC [47]. Subsequently, the gradual salt accumulation damages plant cell membrane which results in ion leakage and lower MSI value [47,80].

Relative water content (RWC) is a parameter to measure plant water status in terms of the physiological consequence of cellular water deficit [81]. Our results showed that salt stress reduced RWC of tomato leaves. A 27% reduction in leaf RWC was observed in tomato plants exposed to 100 mM NaCl [80]. At 150 mM NaCl, tomato inoculated with *D. abyssi* MT1.1^T^ showed 26% reduction in RWC as well as non-inoculated tomato under salt stress. A similar observation was reported from tomato leaves inoculated with *D. barathri* MT2.1^T^ [17]. These findings suggested that salt stress mitigation in deep-sea *Dermacoccus* did not reflect as RWC value. We hypothesized that the mitigation mechanism is more related to osmoregulation as exemplified by the detection of genes related with osmotic stress response in *D. abyssi* MT1.1^T^, in particular osmoprotectant production.

Osmoprotectants, or compatible solutes, are molecules produced by living organisms in response to several abiotic stresses including salinity [82]. Accumulation of compatible solutes is the mechanism for osmoregulation under salt stress in plants [5,10] and microorganisms [27,83]. They can be divided into four main classes; (1) the N-containing solutes such as proline and glycine betaine; (2) sugars such as sucrose and raffinose; (3) straight-chain polyhydric alcohols (polyols) such as mannitol and sorbitol; and (4) cyclic polyhydric alcohols (cyclic polyols) [5]. *D. abyssi* MT1.1^T^ genome contained genes related to compatible solutes (ectoine, choline, and betaine) biosynthesis and regulation (Table 2). Ectoine and its derivatives hydroxyectoine are regarded as N-containing solutes, which were accumulated in response to high salinity and temperature by many microorganisms including actinobacteria [84,85]. When exposed to salt stress (high-osmolarity) environments microorganisms accumulated compatible solutes to increase osmotic potential with in the cells to balance osmotic gradient across the cytoplasmic membrane [85]. Intracellular ectoine was detected in *Streptomyces coelicolor* A3(2) growing in culture broth supplemented with 0.5 M NaCl [86]. In addition, genes involved in choline and betaine uptake and betaine biosynthesis were observed. Glycine betaine can be taken up directly from the environment by specific transport systems or synthesized from choline [87,88]. Glycine betaine transporter OpuD was found in *D. abyssi* MT1.1^T^; the opuD gene product is important for glycine betaine uptake and osmoprotectant in *Bacillus subtilis* [88]. High-affinity choline uptake protein BetT was also found in *D. abyssi* MT1.1^T^. BetT functions as osmoregulatory protein in choline glycine betaine synthesis pathway [89]. Moreover, choline dehydrogenase and betaine aldehyde dehydrogenase encoding genes, involved in glycine betaine biosynthesis, were also found. The detection of genes related to the production of betaine, choline, and glycine betaine provided supporting evidence that *D. abyssi* MT1.1^T^ might use these compatible solutes for mitigation of salt stress in tomato seedlings.

At 150 mM NaCl, cell membrane damage was evident from the reduction in MSI value of tomato leaves compared to non-inoculated tomato without salt stress. Tomato inoculated with *D. abyssi* MT1.1^T^ showed higher MSI value compared to non-inoculated tomato under salt stress. One benefit of plant growth promoting bacteria inoculation in salt stressed plants is reflected in a higher MSI value as reported in alfalfa plants inoculated with either *Hartmannibacter diazotrophicus* or *Pseudomonas* sp. [90] and tomato inoculated with deep-sea *D. profundi* MT2.2^T^ [17]. However, in some instance, the inoculation of plant growth promoting bacteria may not affect the MSI though it helps to increase overall growth performance (shoot and root length, fresh and dry weight) [17]. These results suggested that MSI restoration may not be the main mechanism of *D. abyssi* MT1.1^T^ to mitigate salt stress in tomato seedlings

Total soluble sugar content was increased with the inoculation of *D. abyssi* MT1.1^T^ compared to non-inoculated tomato without salt stress. This result supported the role of total soluble sugar in osmoregulation of plants under salt stress. A 54% increase in total soluble sugar of tomato inoculated with *D. abyssi* MT1.1^T^ under salt stress was observed. It is likely that *D. abyssi* MT1.1^T^ mitigated salt stress in tomato seedlings via accumulation of total soluble sugar. Previously, the inoculation of other *Dermacoccus* strains, *D. barathri* MT2.1^T^ and *D. profundi* MT2.2^T^, did not result in an increase in total soluble sugar in tomato seedlings under salt stress [17]. This data points out that members of deep-sea *Dermacoccus* species are equipped with different mitigation mechanisms and they are considered a versatile plant growth promoting actinobacteria.

Trehalose is only presented in extremophilic or cryptobiotic organisms [91] and acts as an osmoprotectant to protect cells under abiotic conditions including salt stress [29]. Mining of *D. abyssi* MT1.1^T^ genome predicted the genes encoded protein related to trehalose biosynthesis. Several genes with major roles in three bacterial trehalose biosynthetic pathways were found: (1) trehalose synthase pathway (alpha-amylase, trehalose synthase, and putative glucanase genes), (2) TreY/TreZ pathway (malto-oligosyltrehalose synthase and malto-oligosyltrehalose trehalohydrolase genes), and (3) Trehalose-6-phosphate synthase/trehalose-6-phosphate phosphatase (TPS/TPP) pathway (trehalose-6-phosphate phosphatase gene. Genes involved in trehalose biosynthesis were reported from plant growth promoting bacteria including actinobacteria. High desiccation-tolerant actinobacterium, *Microbacterium* sp. 3J1 was reported to contain genes encoding for alpha, alpha-trehalose-phosphate synthase, and trehalose-6-phosphate phosphatase (*otsAB*). This strain accumulated a high amount of intracellular trehalose concentration which correlated with its ability to protect pepper plants under drought stress as seen from fresh and dry weight, root length, and RWC in inoculated-pepper plants [45]. The introduction of these *otsAB* genes in desiccation-sensitive *Pseudomanas putida* KT2440 resulted in a higher accumulation of intracellular trehalose. The inoculation of this strain improved drought tolerance in pepper plants grown under water deficit condition [45]. Similarly, the introduction of *treS* gene in wild type *Pseudomonas* sp. UW4 resulted in a higher trehalose production and salt tolerance in tomato [32]. The prediction of genes for trehalose production in *D. abyssi* MT1.1^T^ and results from previous studies support the role of trehalose in the mitigation of salt stress in plants.

Proline is an amino acid which plays a major role in stress tolerance in crop plants by acting as an osmolyte and an antioxidant [92,93,94]. It can be accumulated inside the cells at high concentrations without any adverse effects on essential cellular functions [88]. Proline accumulation is a common response of bacteria and plants under salt stress [95]. In this study, proline content in tomato leaves was increased in both inoculated and non-inoculated tomato under salt stress (Figure 2B). The common pathway for proline biosynthesis derives from glutamate [95,96]. Several predictive genes related to proline synthesis via glutamate were found in genome of *D. abyssi* MT1.1^T^ including delta-1-pyrroline-5-carboxylate dehydrogenase, pyrroline-5-carboxylate reductase, gamma-glutamyl phosphate reductase, and glutamate 5-kinase. Genes involved in proline transportation, proline/sodium symporter PutP and L-Proline/Glycine betaine transporter ProP were also detected. The detection of these predictive genes involved in proline biosynthesis clearly indicate the ability of *D. abyssi* MT1.1^T^ to produce proline. However, in tomato inoculated with of *D. abyssi* MT1.1^T^, proline level was lower than non-inoculated tomato under salt stress. A similar observation was found in tomato inoculated with *D. barathri* MT2.1^T^ [17], *Streptomyces* sp. PGPA39 [14], maize inoculated with *Azotobacter* sp. C5 and C5 [97], and alfalfa inoculated and co-inoculated with *Arthrobacter* strains MS-1, MS-2, and symbiotic bacteria (*Sinorhizobium meliloti* strains R1 and R2) [22]. Interestingly, plants inoculated with these actinobacteria showed growth performance that was not impaired by salt stress, in particular the dry weight and chlorophyll content. However, a higher proline accumulation in plants under salt stress was also reported in another plant growth promoting bacteria. For example, mungbean inoculated with *Bacillus cereus* Pb25 showed an increase in salt tolerance as a result of a higher proline level as compared to non-inoculated control under 9 dS m^−1^ NaCl [98]. Similarly, drought tolerance in plants inoculated with plant growth promoting bacteria can be either positively correlated or negatively correlated with proline accumulation as exemplified by reports of [37,99,100]. This accumulating evidence points to a challenging question regarding the role of proline in mitigation of abiotic stress in plants by plant growth promoting bacteria.

Salt stress is an oxidative stress which leads to the generation of reactive oxygen species (ROS) in plants [13]. The excessive accumulation of ROS disturbs cell homeostasis such as enzyme inhibition, lipid peroxidation, nucleic acid damage, protein oxidation, and program cell death activation [5,13]. Hydrogen peroxide (H_2_O_2_) is the most important non-radical ROS and a good marker for oxidative stress [30]. Approximately, 2.9 times increased in H_2_O_2_ content was observed in non-inoculated tomato leaves under salt stress compared to non-inoculated tomato without salt stress. Interestingly, inoculation of *D. abyssi* MT1.1^T^ resulted in 78% decreased in H_2_O_2_ content compared to non-inoculated tomato under salt stress. The detoxification of ROS is generally achieved by two mechanisms: enzymatic and non-enzymatic antioxidative systems [30]. *D. abyssi* MT1.1^T^ is an obligate aerobic and catalase producing Gram positive bacteria [34]. Our results suggested that *D. abyssi* MT1.1^T^ may produce antioxidative enzymes such as catalase (CAT) and peroxidase (POD) enzymes to scavenge H_2_O_2_. Similarly, inoculation of deep-sea *D. barathri* MT2.1^T^ and *D. profundi* MT2.2^T^ decreased H_2_O_2_ content in tomato under 150 mM NaCl [17]. Inoculation of *B. cereus* Pb25 reduced H_2_O_2_ and superoxide dismutase (SOD) in salt stressed mungbean leaves corresponded to an increase in catalase (CAT) and peroxidase (POD) activities in leaves [98]. Tomato plants inoculated with *B. velezensis* FMH2 under 60, 120, and 171 mM NaCl was reduced in H_2_O_2_, and malondialdehyde (MDA) content due to an improved phenol peroxidase (POX) activity as compared to non-inoculated tomato plants [101]. Our results and the data from the literature point out that H_2_O_2_ reduction is one of the strategies plant growth promoting bacteria including *Dermacoccus* strains used to mitigate salt stress in plants.

In addition, *D. abyssi* MT1.1^T^ contained predictive genes involved in oxidative stress protective system: superoxide dismutase, alkyl hydroperoxide reductase subunit C (*Ahp*C), and glutathionylspermidine synthase (*Gss*). Superoxide dismutase is responsible for the conversion of the highly reactive superoxide anion to O_2_ and to the less reactive species H_2_O_2_. The AhpC is responsible for the detoxification of reactive oxygen species (including H_2_O_2_) that form in bacterial cells [102,103]. Glutathionylspermidine is a member of low molecular weight thiol antioxidants which found in many Gram positive bacteria including actinobacteria [104]. This protein produce by Gss enzyme from glutathione and spermidine [105]. Both experimental and genome analysis data suggested that *D. abyssi* MT1.1^T^ reduced oxidative damage from salt stress in tomato seedlings by H_2_O_2_ scavenging ability via both enzymatic (catalase) and non-enzymatic (glutathionylspermidine) antioxidative system.

Photosynthetic system is negatively affected by salt stress through chlorophyll content reduction, reflected in plants by poor growth and reduced overall productivity [3,11,106]. Higher concentrations of H_2_O_2_ is reported to reduce chlorophyll content in plants such as tomato [107] and soursop [108]. A 64% reduction in total chlorophyll content of tomato leaves under salt stress was observed. The inoculation of *D. abyssi* MT1.1^T^ increased total chlorophyll content in tomato seedlings compared to non-inoculated tomato under salt stress. These results suggested that photosynthesis of tomato seedlings was improved by the inoculation of *D. abyssi* MT1.1^T^ due to the reduction in H_2_O_2_ induced under salt stress. This increased chlorophyll content corresponded well with the total dry weight observed in tomato-inoculated with *D. abyssi* MT1.1^T^ under salt stress. These results are consistent with previous findings that inoculation of plant growth promoting actinobacteria increases chlorophyll content and growth performance of plants, for example tomato inoculated with *D. barathri* MT2.1^T^ and *D. profundi* MT2.2^T^ [17], *Streptomyces* sp. strain PGPA39 [14], and *Pseudomonas* sp. strain UW4 [109]. Similar observations were recorded in soybean inoculated with *Arthrobacter woluwensis* AK1 [18], *Bacillus aryabhattai* ALT29 [19] and *Arthrobacter woluwensis* ALT43 [19], and alfalfa co-inoculated of *Arthrobacter* strain M1, M2, and *Nocardiopsis* Ag-1 with rhizobium *Sinorhizobium meliloti* R1 and R2 [22].

### 4.4. Biosafety Test for Actinobacteria

Biosafety of potential plant growth promoting bacteria for use as bioinoculants is of general concern. In this study, we evaluated the pathogenicity of *D. abyssi* MT1.1^T^ on *C. elegans* and the potential impacts of the products released by *D. abyssi* MT1.1^T^ on *E. coli* MC4100 viability. Nematode *C. elegans* is an invertebrate model to study the pathogenicity of infectious bacteria in mammals, including humans, as its genome contained at least 36% of genes encoded for protein similar to human [110]. In addition, this nematode is widely used as a model for studying human diseases [111,112,113], aging [114], and pathogenesis of human pathogens [115]. Recently, *C. elegans* was also used as a model organism for evaluating the neuroprotective and neurotherapeutic potential and safety of nutraceuticals in humans [116]. In addition, the biosafety test on *C. elegans* is widely accepted by the scientific community [117,118,119].

In this study, the effect of deep-sea *D. abyssi* MT1.1^T^ on adults, juveniles, eggs, and death rate of *C. elegans* was observed. As expected, the lowest death (1.6%) was found in non-pathogenic strain (*E. coli* OP50) whereas the highest death (100%) was observed in nematodes fed with pathogenic strain (*P. aeruginosa* PA14). A 5% death rate was recorded from *C. elegans* fed with *D. abyssi* MT1.1^T^. Previously, the death rate of nematodes fed with other members of genus *Dermacoccus*, *D. barathri* MT2.1^T^ (1.8%), *D. profundi* MT2.2^T^ (11%), and *D. nishinomiyaensis* DSM20448^T^ (23%), was reported [17]. These observations at least supported the safety of *D. abyssi* MT1.1^T^ as biostimulants in comparison with *D. profundi* MT2.2^T^ and *D. nishinomiyaensis* DSM20448^T^.

In addition, a higher survival percentage of *E. coli* MC4100 was observed in cells exposed to supernatant of *D. abyssi* MT1.1^T^ (99%) compared with non-pathogenic strain, *P. putida* KT2440 (92%). This percentage of survival is the highest among members of the genus *Dermacoccus*; *D. barathri* MT2.1^T^ (92%), *D. profundi* MT2.2^T^ (87%), and *D. nishinomiyaensis* DSM20448^T^ (62%) [17]. These results indicate that the released secondary metabolites from *D. abyssi* MT1.1^T^ should not have deleterious effects on a bacterial community in the environment. In silico analysis of *D. abyssi* MT1.1^T^ genome using PathogenFinder 1.1 confirmed the safety of this strain, as no match was found with any known pathogenic protein families.

In addition, *D. abyssi* MT1.1^T^ is a strain of species and recognized as a risk group 1 microorganism according to the German Technical Rules for Biological Agents (TRBA). The search through the Online Risk Group Database provided by ABSA International (The Association for Biosafety and Biosecurity) found no record regarding the risk of *D. abyssi* MT1.1^T^ as animal, human, or plant pathogens and select agents of both the Center for Disease Control (CDC) and United States Department of Agriculture (USDA). Moreover, both Scopus and Web of Science searches using the keyword “*D. abyssi* MT1.1^T^” found no publication related to negative impact of *D. abyssi* MT1.1^T^ on human or environmental health. This information suggests the safety of *D. abyssi* MT1.1^T^ for humans and the environment. However, further experimental investigations would provide a more concrete supporting evidence on this aspect.

## 5. Conclusions

Our results provide further supporting evidence on the potential of deep-sea actinobacteria to promote the growth of plants under salt stress, in particular members of the genus *Dermacoccus.* The inoculation of *D. abyssi* MT1.1^T^ alleviated salt stress and promoted growth of tomato seedlings under 150 mM NaCl. We proposed that *D. abyssi* MT1.1^T^ mitigated salt stress in tomato seedlings via osmoregulation by the production of compatible solutes (total soluble sugar, proline, glycine betaine, and trehalose) and the reduction in H_2_O_2_ based on experimental and/or genomic analysis data. Survival and colonization of tomato roots by *D. abyssi* MT1.1^T^ were confirmed by a culture-based approach and SEM analysis. The safety of this strain was demonstrated based on bioassay and in silico analysis. In conclusion, this study highlights the suitability of *D. abyssi* MT1.1^T^ as a candidate for the formulation of bioinoculant which offers an alternative for sustainable management of plants under salt-affected area.

## Figures and Tables

**Table 1:** Plant growth promoting activity of *D. abyssi* MT1.1^T^ under different level of salt stress.

NaCl Concentration (mM)	IAA Production (µg mL^−1^)	Siderophore Production (µmol mL^−1^)		Phosphate Solubilization	
		Hydroxamate	Catecholate	P Released in PVK Broth (µg mL^−1^)	pH
0	37.50 ^a^ ± 1.61	46.67 ^a^ ± 17.56	2.98 ^a^ ± 2.90	71.62 ^a^ ± 3.02	5.1 ± 0.07
150	11.75 ^b^ ± 0.35	173.33 ^b^ ± 31.66	48.25 ^b^ ± 17.87	67.98 ^ab^ ± 1.41	5.4 ± 0.03
300	8.36 ^b^ ± 0.32	189.17 ^b^ ± 31.75	21.58 ^c^ ± 2.73	67.09 ^b^ ± 1.79	5.4 ± 0.04
450	10.55 ^b^ ± 5.39	170.83 ^b^ ± 3.82	20.70 ^c^ ± 1.69	62.33^c^ ± 2.13	5.4 ± 0.05

Data represent mean values of three replicates ± SD. The different letters (^a^, ^b^, and ^c^) indicate a significant different in plant growth promoting traits in each NaCl concentration according to Duncan at *p* < 0.05.

**Table 2:** Protein coding sequences related with plant growth promoting traits of *D. abyssi* MT1.1^T^.

PGP Traits	Protein Coding Sequences Conferring PGP Traits
Amino Acids and Derivatives	Proline synthesis: Pyrroline-5-carboxylate reductase (EC 1.5.1.2) Gamma-glutamyl phosphate reductase (EC 1.2.1.41) NADP-specific glutamate dehydrogenase (EC 1.4.1.4) RNA-binding C-terminal domain PUA Glutamate 5-kinase (EC 2.7.2.11) Proline, 4-hydroxyproline uptake and utilization: Proline/sodium symporter PutP (TC 2.A.21.2.1) L-Proline/Glycine betaine transporter ProP Delta-1-pyrroline-5-carboxylate dehydrogenase (EC 1.2.1.88)
	Tryptophan synthesis: Anthranilate synthase, amidotransferase component (EC 4.1.3.27) Aminodeoxychorismate lyase (EC 4.1.3.38) Tryptophan synthase alpha chain (EC 4.2.1.20) Anthranilate phosphoribosyltransferase (EC 2.4.2.18) Tryptophan synthase beta chain (EC 4.2.1.20) Acting phosphoribosylanthranilate isomerase (EC 5.3.1.24) Indole-3-glycerol phosphate synthase (EC 4.1.1.48)
	8. Anthranilate synthase, aminase component (EC 4.1.3.27) 9. Para-aminobenzoate synthase, aminase component (EC 2.6.1.85) 10. Para-aminobenzoate synthase, amidotransferase component (EC 2.6.1.85)
Iron acquisition and metabolism	Ferrous iron transporter EfeUOB, low-pH-induced: Ferrous iron transport peroxidase EfeB Ferrous iron transport permease EfeU Ferrous iron transport periplasmic protein EfeO, contains peptidase-M75 domain and (frequently) cupredoxin-like domain Encapsulating protein for DyP-type peroxidase and ferritin-like protein oligomers: Predicted dye-decolorizing peroxidase (DyP), YfeX-like subgroup
Phosphorus metabolism	High affinity phosphate transporter and control of PHO regulon: Phosphate regulon transcriptional regulatory protein PhoB (SphR) Polyphosphate kinase (EC 2.7.4.1) Phosphate transport system regulatory protein PhoU Phosphate regulon sensor protein PhoR (SphS) (EC 2.7.13.3) Phosphate metabolism: Secreted alkaline phosphatase Phosphate regulon transcriptional regulatory protein PhoB (SphR) Inorganic pyrophosphatase (EC 3.6.1.1) Exopolyphosphatase (EC 3.6.1.11) Phosphate transport system regulatory protein PhoU Probable low-affinity inorganic phosphate transporter Predicted ATPase related to phosphate starvation-inducible protein PhoH Polyphosphate kinase (EC 2.7.4.1) Phosphate starvation-inducible protein PhoH, predicted ATPase Phosphate regulon sensor protein PhoR (SphS) (EC 2.7.13.3) Polyphosphate: Polyphosphate glucokinase (EC 2.7.1.63) Polyphosphate kinase (EC 2.7.4.1) Exopoly phosphatase (EC 3.6.1.11) Polyphosphate kinase 2 (EC 2.7.4.1)
N_2_ metabolism	Ammonia assimilation: Ferredoxin-dependent glutamate synthase (EC 1.4.7.1) Nitrogen regulatory protein P-II Glutamate-ammonia-ligase adenylyltransferase (EC 2.7.7.42) Ammonium transporter Glutamate synthase [NADPH] large chain (EC 1.4.1.13) Glutamine synthetase type I (EC 6.3.1.2) [Protein-PII] uridylyltransferase (EC 2.7.7.59) Glutamate synthase [NADPH] small chain (EC 1.4.1.13)
Trehalose metabolism	Trehalose biosynthesis: Alpha-amylase (EC 3.2.1.1) Malto-oligosyltrehalose synthase (EC 5.4.99.15) 1,4-alpha-glucan (glycogen) branching enzyme, GH-13-type (EC 2.4.1.18) Trehalose synthase (EC 5.4.99.16) Trehalose-6-phosphate phosphatase (EC 3.1.3.12) Putative glucanase glgE (EC 3.2.1.-) Malto-oligosyltrehalose trehalohydrolase (EC 3.2.1.141) Glucoamylase (EC 3.2.1.3)
Potassium metabolism	Potassium homeostasis: Potassium efflux system KefA protein Large-conductance mechanosensitive channel Potassium channel protein Kup system potassium uptake protein
Osmotic stress response	Osmoregulation: Glycerol uptake facilitator proteinEctoine biosynthesis and regulation: L-ectoine synthase (EC 4.2.1.-)Choline and Betaine Uptake and Betaine Biosynthesis: Glycine betaine transporter OpuD High-affinity choline uptake protein BetT Choline dehydrogenase (EC 1.1.99.1) Betaine aldehyde dehydrogenase (EC 1.2.1.8)
Oxidative stress response	Alkyl hydroperoxide reductase subunit C-like proteinGlutathionylspermidine and Trypanothione: Similarity with glutathionylspermidine synthase (EC 6.3.1.8), group 1

## Data Availability

Data sharing not applicable to this article as no datasets were generated or analyzed during the current study.

## Supplementary Materials

The following are available online at https://www.mdpi.com/article/10.3390/biology11020191/s1, Figure S1: Overviews of all experimental methods used in this study, Figure S2: Subsystems of predicted genes in *D. abyssi* MT1.1^T^ genome using RAST server.

## References

[1] Shrivastava P., Kumar R. (2015). Soil salinity : A serious environmental issue and plant growth promoting bacteria as one of the tools for its alleviation. Saudi J. Biol. Sci..

[2] Butcher K., Wick A.F., Desutter T., Chatterjee A., Harmon J. (2016). Soil salinity: A threat to global food security. Agron. J..

[3] Hasanuzzaman M., Nahar K., Alam M.M., Bhowmik P.C., Hossain M.A., Rahman M.M., Prasad M.N.V., Ozturk M., Fujita M. (2014). Potential use of halophytes to remediate saline soils. Biomed Res. Int..

[4] Wang W., Vinocur B., Altman A. (2003). Plant responses to drought, salinity and extreme temperatures: Towards genetic engineering for stress tolerance. Planta.

[5] Parihar P., Singh S., Singh R., Singh V., Prasad S. (2015). Effect of salinity stress on plants and its tolerance strategies: A review. Environ. Sci. Pollut. Res..

[6] Javed Q., Azeem A., Jabran K., Kumar A. (2019). Impacts of salt stress on the physiology of plants and opportunity to rewater the stressed plants with diluted water: A review. Appl. Ecol. Environ. Res..

[7] Reza Yousefi A., Rashidi S., Moradi P., Mastinu A. (2020). Germination and Seedling Growth Responses of Zygophyllum fabago, Salsola kali L. and Atriplex canescens to PEG-Induced Drought Stress. Environments.

[8] Acosta-Motos J., Ortuño M., Bernal-Vicente A., Diaz-Vivancos P., Sanchez-Blanco M., Hernandez J. (2017). Plant responses to salt stress: Adaptive mechanisms. Agronomy.

[9] Hanin M., Ebel C., Ngom M., Laplaze L., Masmoudi K. (2016). New insights on plant salt tolerance mechanisms and their potential use for breeding. Front. Plant Sci..

[10] Munns R., Tester M. (2008). Mechanisms of Salinity Tolerance. Annu. Rev. Plant Biol..

[11] Kamran M., Parveen A., Ahmar S., Hussain S., Chattha M.S., Saleem M.H., Adil M., Heidari P., Chen J. (2020). An overview of hazardous impacts of soil salinity in crops, tolerance mechanisms, and amelioration through selenium supplementation. Int. J. Mol. Sci..

[12] Numan M., Bashir S., Khan Y., Mumtaz R., Shinwari Z.K., Khan A.L., Khan A., AL-Harrasi A. (2018). Plant growth promoting bacteria as an alternative strategy for salt tolerance in plants: A review. Microbiol. Res..

[13] Sharma P., Jha A.B., Dubey R.S., Pessarakli M. (2012). Reactive Oxygen Species, Oxidative Damage, and Antioxidative Defense Mechanism in Plants under Stressful Conditions. J. Bot..

[14] Palaniyandi S.A., Damodharan K., Yang S.H., Suh J.W. (2014). Streptomyces sp. strain PGPA39 alleviates salt stress and promotes growth of “Micro Tom” tomato plants. J. Appl. Microbiol..

[15] Gong Y., Chen L.J., Pan S.Y., Li X.W., Xu M.J., Zhang C.M., Xing K., Qin S. (2020). Antifungal potential evaluation and alleviation of salt stress in tomato seedlings by a halotolerant plant growth-promoting actinomycete *Streptomyces* sp. KLBMP5084. Rhizosphere.

[16] Xiong Y.W., Gong Y., Li X.W., Chen P., Ju X.Y., Zhang C.M., Yuan B., Lv Z.P., Xing K., Qin S. (2019). Enhancement of growth and salt tolerance of tomato seedlings by a natural halotolerant actinobacterium *Glutamicibacter halophytocola* KLBMP 5180 isolated from a coastal halophyte. Plant Soil.

[17] Rangseekaew P., Barros-Rodríguez A., Pathom-Aree W., Manzanera M. (2021). Deep-sea actinobacteria mitigate salinity stress in tomato seedlings and their biosafety testing. Plants.

[18] Khan M.A., Ullah I., Waqas M., Hamayun M., Khan A.L., Asaf S., Kang S., Kim K., Jan R., Lee I. (2019). Halo-tolerant rhizospheric *Arthrobacter woluwensis* AK1 mitigates salt stress and induces physio-hormonal changes and expression of GmST1 and GmLAX3 in soybean. Symbiosys.

[19] Khan M.A., Sahile A.A., Jan R., Asaf S., Hamayun M., Imran M., Adhikari A., Kang S., Kim K., Lee I. (2021). Halotolerant bacteria mitigate the effects of salinity stress on soybean growth by regulating secondary metabolites and molecular responses. BMC Plant Biol..

[20] Sadeghi A., Karimi E., Dahaji P.A., Javid M.G., Dalvand Y., Askari H. (2012). Plant growth promoting activity of an auxin and siderophore producing isolate of Streptomyces under saline soil conditions. World J. Microbiol. Biotechnol..

[21] Djebaili R., Pellegrini M., Rossi M., Forni C., Smati M., Del Gallo M., Kitouni M. (2021). Characterization of plant growth-promoting traits and inoculation effects on triticum durum of actinomycetes isolates under salt stress conditions. Soil Syst..

[22] Saidi S., Cherif H., Ali S., Bouket C., Silini A., Eshelli M., Luptakova L. (2021). Improvement of *Medicago sativa* Crops Productivity by the Co-inoculation of *Sinorhizobium meliloti*–Actinobacteria Under Salt Stress. Curr. Microbiol..

[23] Mathew B.T., Torky Y., Amin A., Mourad A.H.I., Ayyash M.M., El-Keblawy A., Hilal-Alnaqbi A., AbuQamar S.F., El-Tarabily K.A. (2020). Halotolerant Marine Rhizosphere-Competent Actinobacteria Promote Salicornia bigelovii Growth and Seed Production Using Seawater Irrigation. Front. Microbiol..

[24] Suksaard P., Pathom-aree W., Duangmal K. (2017). Diversity and plant growth promoting activities of actinomycetes from mangroves. Chiang Mai J. Sci..

[25] Sathya A., Vijayabharathi R., Gopalakrishnan S. (2017). Plant growth-promoting actinobacteria: A new strategy for enhancing sustainable production and protection of grain legumes. 3 Biotech.

[26] Glick B.R. (2012). Plant growth-promoting bacteria: Mechanisms and applications. Scientifica.

[27] Sagar A., Rai S., Ilyas N., Sayyed R.Z., Al-Turki A.I., El Enshasy H.A., Simarmata T. (2022). Halotolerant Rhizobacteria for Salinity-Stress Mitigation: Diversity, Mechanisms and Molecular Approaches. Sustainability.

[28] Vílchez J.I., Niehaus K., Dowling D.N., González-López J., Manzanera M. (2018). Protection of Pepper Plants from Drought by *Microbacterium* sp. 3J1 by Modulation of the Plant’s Glutamine and α-ketoglutarate Content: A Comparative Metabolomics Approach. Front. Microbiol..

[29] Sharma A., Shahzad B., Kumar V., Kohli S.K., Sidhu G.P.S., Bali A.S., Handa N., Kapoor D., Bhardwaj R., Zheng B. (2019). Phytohormones regulate accumulation of osmolytes under abiotic stress. Biomolecules.

[30] Sofo A., Scopa A., Nuzzaci M., Vitti A. (2015). Ascorbate peroxidase and catalase activities and their genetic regulation in plants subjected to drought and salinity stresses. Int. J. Mol. Sci..

[31] Kumar A., Singh S., Gaurav A.K., Srivastava S. (2020). Plant Growth-Promoting Bacteria : Biological Tools for the Mitigation of Salinity Stress in Plants. Front. Microbiol..

[32] Orozco-mosqueda C., Glick B.R., Santoyo G. (2020). ACC deaminase in plant growth-promoting bacteria (PGPB): An e ffi cient mechanism to counter salt stress in crops. Microbiol. Res..

[33] Barros-Rodríguez A., Rangseekaew P., Lasudee K., Pathom-aree W., Manzanera M. (2020). Regulatory risks associated with bacteria as biostimulants and biofertilizers in the frame of the European Regulation (EU) 2019/1009. Sci. Total Environ..

[34] Pathom-Aree W., Nogi Y., Sutcliffe I.C., Ward A.C., Horikoshi K., Bull A.T., Goodfellow M. (2006). Dermacoccus abyssi sp. nov., a piezotolerant actinomycete isolated from the Mariana Trench. Int. J. Syst. Evol. Microbiol..

[35] Abdel-Mageed W.M., Juhasz B., Lehri B., Alqahtani A.S., Nouioui I., Pech-Puch D., Tabudravu J.N., Goodfellow M., Rodríguez J., Jaspars M. (2020). Whole genome sequence of dermacoccus abyssi MT1.1 isolated from the challenger deep of the mariana trench reveals phenazine biosynthesis locus and environmental adaptation factors. Mar. Drugs.

[36] Pathom-aree W., Nogi Y., Ward A.C., Horikoshi K., Bull A.T., Goodfellow M. (2006). Dermacoccus barathri sp. nov. and Dermacoccus profundi sp. nov., novel actinomycetes isolated from deep-sea mud of the Mariana Trench. Int. J. Syst. Evol. Microbiol..

[37] Lasudee K., Tokuyama S., Lumyong S., Pathom-Aree W. (2018). Actinobacteria Associated with arbuscular mycorrhizal funneliformis mosseae spores, taxonomic characterization and their beneficial traits to plants: Evidence obtained from mung bean (*Vigna radiata*) and Thai Jasmine Rice (*Oryza sativa*). Front. Microbiol..

[38] Schwyn B., Neilands J.B. (1987). Universal chemical assay for the detection and determination of siderophores. Anal. Biochem..

[39] Djebaili R., Pellegrini M., Smati M., Del Gallo M., Kitouni M. (2020). Actinomycete strains isolated from saline soils: Plant-growth-promoting traits and inoculation effects on solanum lycopersicum. Sustainability.

[40] Overbeek R., Olson R., Pusch G.D., Olsen G.J., Davis J.J., Disz T., Edwards R.A., Gerdes S., Parrello B., Shukla M. (2014). The SEED and the Rapid Annotation of microbial genomes using Subsystems Technology (RAST). Nucleic Acids Res..

[41] Blin K., Shaw S., Kloosterman A.M., Charlop-Powers Z., van Wezel G.P., Medema M.H., Weber T. (2021). AntiSMASH 6.0: Improving cluster detection and comparison capabilities. Nucleic Acids Res..

[42] Skinnider M.A., Merwin N.J., Johnston C.W., Magarvey N.A. (2017). PRISM 3: Expanded prediction of natural product chemical structures from microbial genomes. Nucleic Acids Res..

[43] Cosentino S., Voldby Larsen M., Møller Aarestrup F., Lund O. (2013). PathogenFinder - Distinguishing friend from foe using bacterial whole genome sequence data. PLoS ONE.

[44] Narváez-Reinaldo J.J., Barba I., González-López J., Tunnacliffe A., Manzanera M. (2010). Rapid Method for Isolation of Desiccation-Tolerant Strains and Xeroprotectants. Appl. Environ. Microbiol..

[45] Vílchez J.I., García-Fontana C., Román-Naranjo D., González-López J., Manzanera M. (2016). Plant drought tolerance enhancement by trehalose production of desiccation-tolerant microorganisms. Front. Microbiol..

[46] Oukarroum A., El Madidi S., Schansker G., Strasser R.J. (2007). Probing the responses of barley cultivars (*Hordeum vulgare* L.) by chlorophyll a fluorescence OLKJIP under drought stress and re-watering. Environ. Exp. Bot..

[47] Ashraf M., Shahzad S.M., Akhtar N., Imtiaz M., Ali A. (2017). Salinization/sodification of soil and physiological dynamics of sunflower irrigated with saline–sodic water amending by potassium and farm yard manure. J. Water Reuse Desalin..

[48] Arnon D.I. (1949). Copper enzymes in isolated chloroplasts. polyphenoloxidase in Beta vulgaris. Plant Physiol..

[49] Bates L.S., Waldren R.P., Teare I.D. (1973). Rapid determination of free proline for water-stress studies. Plant Soil.

[50] DuBois M., Gilles K.A., Hamilton J.K., Rebers P.A., Smith F. (1956). Colorimetric method for determination of sugars and related substances. Anal. Chem..

[51] Velikova V., Yordanov I., Edreva A. (2000). Oxidative stress and some antioxidant systems in acid rain-treated bean plants. Plant Sci..

[52] Botta A.L., Santacecilia A., Ercole C., Cacchio P., Del Gallo M. (2013). In vitro and in vivo inoculation of four endophytic bacteria on Lycopersicon esculentum. N. Biotechnol..

[53] Vílchez J.I., Navas A., González-lópez J., Arcos S.C., Gutierrez F.J. (2016). Biosafety test for plant growth-promoting bacteria: Proposed Environmental and Human Safety Index (EHSI) protocol. Front. Microbiol..

[54] Vílchez S., Tunnacliffe A., Manzanera M. (2008). Tolerance of plastic-encapsulated Pseudomonas putida KT2440 to chemical stress. Extremophiles.

[55] García-Fontana C., Vílchez J.I., González-Requena M., González-López J., Krell T., Matilla M.A., Manzanera M. (2019). The involvement of McpB chemoreceptor from Pseudomonas aeruginosa PAO1 in virulence. Sci. Rep..

[56] Hoelzle K., Peter S., Sidler M., Kramer M.M., Wittenbrink M.M., Felder K.M., Hoelzle L.E. (2010). Inorganic pyrophosphatase in uncultivable hemotrophic mycoplasmas: Identification and properties of the enzyme from Mycoplasma suis. BMC Microbiol..

[57] Bhatti A.A., Haq S., Bhat R.A. (2017). Actinomycetes benefaction role in soil and plant health. Microb. Pathog..

[58] Tsavkelova E.A., Klimova S.Y., Cherdyntseva T.A., Netrusov A.I. (2006). Microbial producers of plant growth stimulators and their practical use: A review. Appl. Biochem. Microbiol..

[59] Wang W., Qiu Z., Tan H., Cao L. (2014). Siderophore production by actinobacteria. BioMetals.

[60] Alori E.T., Glick B.R., Babalola O.O. (2017). Microbial phosphorus solubilization and its potential for use in sustainable agriculture. Front. Microbiol..

[61] Zhu F., Qu L., Hong X., Sun X. (2011). Isolation and characterization of a phosphate- solubilizing halophilic bacterium *Kushneria* sp. YCWA18 from Daqiao Saltern on the coast of yellow sea of China. Evid. Based Complement. Altern. Med..

[62] Sharma S.B., Sayyed R.Z., Trivedi M.H., Gobi T.A. (2013). Phosphate solubilizing microbes: Sustainable approach for managing phosphorus deficiency in agricultural soils. Springerplus.

[63] Ahemad M., Kibret M. (2014). Mechanisms and applications of plant growth promoting rhizobacteria: Current perspective. J. King Saud Univ.-Sci..

[64] Spaepen S., Vanderleyden J., Remans R. (2007). Indole-3-acetic acid in microbial and microorganism-plant signaling. FEMS Microbiol. Rev..

[65] Insuk C., Kuncharoen N., Cheeptham N., Tanasupawat S., Pathom-aree W. (2020). Bryophytes harbor cultivable actinobacteria with plant growth promoting potential. Front. Microbiol..

[66] Kaewkla O., Suriyachadkun C., Milton C., Franco M. (2021). Streptomyces adelaidensis sp. nov., an actinobacterium isolated from the root of Callitris preissii with potential for plant growth-promoting properties. Arch. Microbiol..

[67] Subramaniam G., Thakur V., Saxena R.K., Vadlamudi S., Purohit S., Kumar V., Rathore A., Chitikineni A., Varshney R.K. (2020). Complete genome sequence of sixteen plant growth promoting Streptomyces strains. Sci. Rep..

[68] Ouyang J., Shao X., Li J. (2000). Indole-3-glycerol phosphate, a branchpoint of indole-3- acetic acid biosynthesis from the tryptophan biosynthetic pathway in Arabidopsis thaliana. Plant J..

[69] Singh S.P., Gupta R., Gaur R., Srivastava A.K. (2017). Antagonistic Actinomycetes Mediated Resistance in Solanum lycopersicon Mill. Against Rhizoctonia solani Kühn. Proc. Natl. Acad. Sci. India Sect. B-Biol. Sci..

[70] Goudjal Y., Toumatia O., Sabaou N., Barakate M., Mathieu F., Zitouni A. (2013). Endophytic actinomycetes from spontaneous plants of Algerian Sahara: Indole-3-acetic acid production and tomato plants growth promoting activity. World J. Microbiol. Biotechnol..

[71] Andrews S.C., Robinson A.K., Rodríguez-Quiñones F. (2003). Bacterial iron homeostasis. FEMS Microbiol. Rev..

[72] Sandy M., Butler A. (2009). Microbial Iron Acquisition: Marine and Terrestrial Siderophores. Chem. Rev..

[73] Lau C.K.Y., Krewulak K.D., Vogel H.J. (2015). Bacterial ferrous iron transport : The Feo system. FEMS Microbiol. Rev..

[74] Dastager S.G., Damare S. (2013). Marine actinobacteria showing phosphate-solubilizing efficiency in Chorao Island, Goa, India. Curr. Microbiol..

[75] Santos-Beneit F. (2015). The Pho regulon: A huge regulatory network in bacteria. Front. Microbiol..

[76] Martín J.F., Liras P. (2021). Molecular mechanisms of phosphate sensing, transport and signalling in streptomyces and related actinobacteria. Int. J. Mol. Sci..

[77] Rawat P., Das S., Shankhdhar D., Shankhdhar S.C. (2021). Phosphate-solubilizing microorganisms: Mechanism and their role in phosphate solubilization and uptake. J. Soil Sci. Plant Nutr..

[78] Luo H., Benner R., Long R.A., Hu J. (2009). Subcellular localization of marine bacterial alkaline phosphatases. Proc. Natl. Acad. Sci. USA.

[79] Burkovski A. (2003). Ammonium assimilation and nitrogen control in Corynebacterium glutamicum and its relatives: An example for new regulatory mechanisms in actinomycetes. FEMS Microbiol. Rev..

[80] Tanveer K., Gilani S., Hussain Z., Ishaq R., Adeel M., Ilyas N. (2019). Effect of salt stress on tomato plant and the role of calcium. J. Plant Nutr..

[81] Barrs H., Weatherley P. (1962). A re-examination of the relative turgidity technique for estimating water deficits in leaves. Aust. J. Biol. Sci..

[82] Kempf M.B., Bremer E. (1998). Uptake and synthesis of compatible solutes as microbial stress responses to high-osmolality environments. Arch. Microbiol..

[83] León M.J., Hoffmann T., Sánchez-porro C., Heider J., Ventosa A., Bremer E., Oren A. (2018). Compatible solute synthesis and import by the moderate halophile Spiribacter salinus: Physiology and genomics. Front. Microbiol..

[84] Crowley E. (2017). Compatible solute ectoine review: Protection mechanisms and production methods. J. Undergrad. Stud. Trent.

[85] Czech L., Hermann L., Stöveken N., Richter A.A., Id A.H., Smits S.H.J., Heider J., Bremer E. (2018). Role of the extremolytes ectoine and hydroxyectoine as stress protectants and nutrients: Genetics, phylogenomics, biochemistry, and structural analysis. Genes.

[86] Bursy J., Kuhlmann A.U., Pittelkow M., Hartmann H., Jebbar M., Pierik A.J., Bremer E. (2008). Synthesis and uptake of the compatible solutes ectoine and 5-hydroxyectoine by Streptomyces coelicolor A3 (2) in response to salt and heat stresses. Appl. Environ. Microbiol..

[87] Wargo M.J. (2013). Homeostasis and catabolism of choline and glycine betaine: Lessons from Pseudomonas aeruginosa. Appl. Environ. Microbiol..

[88] Kappes R.M., Kempf B., Bremer E. (1996). Three transport systems for the osmoprotectant glycine betaine pperate in Bacillus subtilis: Characterization of OpuD. J. Bacteriol..

[89] Lamark T., Kaasen I., Eshoo M.W., Falkenberg P., Mcdougall J., Strom A.R. (1991). DNA sequence and analysis of the bef genes encoding the osmoregulatory choline-glycine betaine pathway of *Escherichia coli* B. Mol. Microbiol..

[90] Ansari M., Shekari F., Mohammadi M.H., Juhos K., Végvári G., Biró B. (2019). Salt-tolerant plant growth-promoting bacteria enhanced salinity tolerance of salt-tolerant alfalfa (*Medicago sativa* L.) cultivars at high salinity. Acta Physiol. Plant..

[91] Avonce N., Mendoza-vargas A., Morett E., Iturriaga G. (2006). Insights on the evolution of trehalose biosynthesis. BMC Evol. Biol..

[92] Ben Rejeb K., Abdelly C., Savouré A. (2014). How reactive oxygen species and proline face stress together. Plant Physiol. Biochem..

[93] Jamali S.S., Borzouei A., Aghamirzaei M., Khosronejad H.R., Fathi M. (2015). Cell membrane stability and biochemical response of seven wheat cultivars under salinity stress. Rev. Bras. Bot..

[94] Kaushal M., Wani S.P. (2016). Plant-growth-promoting rhizobacteria: Drought stress alleviators to ameliorate crop production in drylands. Ann. Microbiol..

[95] Fichman Y., Gerdes S.Y., Kovács H., Szabados L., Zilberstein A., Csonka L.N. (2015). Evolution of proline biosynthesis: Enzymology, bioinformatics, genetics, and transcriptional regulation. Biol. Rev..

[96] Liang Q.J., Wu X.J., Yang P., Kong J.R., Wei W., Qiao X., Liu Y., Wang W. (2019). The role of delta-1-pyrroline-5-carboxylate dehydrogenase (P5CDh) in the Pacific white shrimp (*Litopenaeus vannamei*) during biotic and abiotic stress. Aquat. Toxicol..

[97] Rojas-tapias D., Moreno-galván A., Pardo-díaz S., Obando M., Rivera D., Bonilla R. (2012). Effect of inoculation with plant growth-promoting bacteria (PGPB) on amelioration of saline stress in maize (*Zea mays*). Appl. Soil Ecol..

[98] Islam F., Yasmeen T., Arif M.S., Ali S. (2016). Plant growth promoting bacteria confer salt tolerance in Vigna radiata by up-regulating antioxidant defense and biological soil fertility. Plant Growth Regul..

[99] Curá J.A., Franz D.R., Filosofía J.E., Balestrasse K.B., Burgueño L.E. (2017). Inoculation with Azospirillum sp. and Herbaspirillum sp. bacteria increases the tolerance of maize to drought stress. Microorganisms.

[100] Abbasi S., Sadeghi A., Safaie N. (2020). Streptomyces alleviate drought stress in tomato plants and modulate the expression of transcription factors ERF1 and WRKY70 genes. Sci. Hortic..

[101] Masmoudi F., Tounsi S., Dunlap C.A., Trigui M. (2021). Endophytic halotolerant Bacillus velezensis FMH2 alleviates salt stress on tomato plants by improving plant growth and altering physiological and antioxidant responses. Plant Physiol. Biochem..

[102] Cha M., Bae Y., Kim K., Park B., Kim I., Cha M., Bae Y., Kim K. (2015). Characterization of two alkyl hydroperoxide reductase C homologs alkyl hydroperoxide reductase C_H1 and alkyl hydroperoxide reductase C_H2 in Bacillus subtilis. World J. Biol. Chem..

[103] Wang H., Chung C., Ma T., Wong H. (2013). Roles of alkyl hydroperoxide reductase subunit C (AhpC) in viable but nonculturable Vibrio parahaemolyticus. Appl. Environ. Microbiol..

[104] Chattopadhyay M.K., Chen W., Tabor H. (2013). Escherichia coli glutathionylspermidine synthetase/amidase: Phylogeny and effect on regulation of gene expression. FEMS Microbiol. Lett..

[105] Kwon D.S., Lin C., Chen S., Coward J.K., Walsh C.T., Bollinger J.M. (1997). Dissection of glutathionylspermidine synthetase/amidase from Escherichia coli into autonomously folding and functional synthetase and amidase domains. J. Biol. Chem..

[106] Machado R., Serralheiro R. (2017). Soil salinity: Effect on vegetable crop growth. management practices to prevent and mitigate soil salinization. Horticulturae.

[107] Al-aghabary K., Zhu Z., Shi Q. (2007). Influence of silicon supply on chlorophyll content, chlorophyll fluorescence, and antioxidative enzyme activities in tomato plants under salt stress antioxidative enzyme activities in tomato. J. Plant Nutr..

[108] Da Silva A.A.R., de Lima G.S., de Azevedo C.A.V., de S.A. Veloso L.L., Gheyi H.R., dos A. Soares L.A. (2019). Salt stress and exogenous application of hydrogen peroxide on photosynthetic parameters of soursop. Rev. Bras. Eng. Agrícola Ambient..

[109] Orozco-mosqueda M.C., Duan J., Dibernardo M., Zetter E., Campos-garcía J., Glick B.R., Santoyo G. (2019). The production of ACC deaminase and trehalose by the plant growth promoting bacterium Pseudomonas sp. UW4 synergistically protect tomato plants against salt stress. Front. Microbiol..

[110] Darby C., Cosma C.L., Thomas J.H., Manoil C. (1999). Lethal paralysis of *Caenorhabditis elegans* by *Pseudomonas aeruginosa*. Proc. Natl. Acad. Sci. USA.

[111] Harrington A.J., Hamamichi S., Caldwell G.A., Caldwell K.A. (2010). *C. elegans* as a model organism to investigate molecular pathways involved with Parkinson’s disease. Dev. Dyn..

[112] Park H.H., Jung Y., Lee S.V. (2017). Molecules and Cells Survival assays using *Caenorhabditis elegans*. Mol. Cells.

[113] Maglioni S., Ventura N. (2016). *C. elegans* as a model organism for human mitochondrial associated disorders. Mitochondrion.

[114] Zhang S., Li F., Zhou T., Wang G., Li Z. (2020). *Caenorhabditis elegans* as a Useful Model for Studying Aging Mutations. Front. Endocrinol..

[115] Marsh E.K., May R.C. (2012). *Caenorhabditis elegans*, A model organism for investigating immunity. Appl. Environ. Microbiol..

[116] Darvesh A.S., Barnett R.E., Fitsanakis V.A., Gupta R.C., Lall R., Srivastava A. (2021). *Caenorhabditis elegans*: An elegant model organism for evaluating the neuroprotective and neurotherapeutic potential of nutraceuticals. Nutraceuticals.

[117] De Sousa Figueiredo M.B., Pradel E., George F., Mahieux S., Houcke I., Pottier M., Fradin C., Neut C., Daniel C., Bongiovanni A. (2021). Adherent-Invasive and Non-Invasive *Escherichia coli* Isolates Differ in Their Effects on *Caenorhabditis elegans*’ Lifespan. Microorganisms.

[118] Sánchez-Diener I., Zamorano L., López-Causapé C., Cabot G., Mulet X., Peña C., del Campo R., Cantón R., Doménech-Sánchez A., Martínez-Martínez L. (2017). Interplay among Resistance Profiles, High-Risk Clones, and Virulence in the *Caenorhabditis elegans*
*Pseudomonas aeruginosa* Infection Model. Antimicrob. Agents Chemother..

[119] Keswani C., Prakash O., Bharti N., Vílchez J.I., Sansinenea E., Lally R.D., Borriss R., Singh S.P., Gupta V.K., Fraceto L.F. (2019). Re-addressing the biosafety issues of plant growth promoting rhizobacteria. Sci. Total Environ..

