# Supplementary material for: Plant Beneficial Deep-Sea Actinobacterium, Dermacoccus abyssi MT1.1T Promote Growth of Tomato (Solanum lycopersicum) under Salinity Stress
Source: Biology (Basel). 2022 Jan 26;11(2):191. doi: 10.3390/biology11020191 (PMC8869415; doi:10.3390/biology11020191)
Supplement: Supplementary file 1 [file biology-11-00191-s001.zip › biology-1559843-Supplementary materials.pdf]

Supplementary figures for "Plant Beneficial Deep-Sea Actinobacterium, *Dermacoccus abyssi* MT1.1ᵀ Promote Growth of Tomato (*Solanum lycopersicum*) under Salinity Stress"

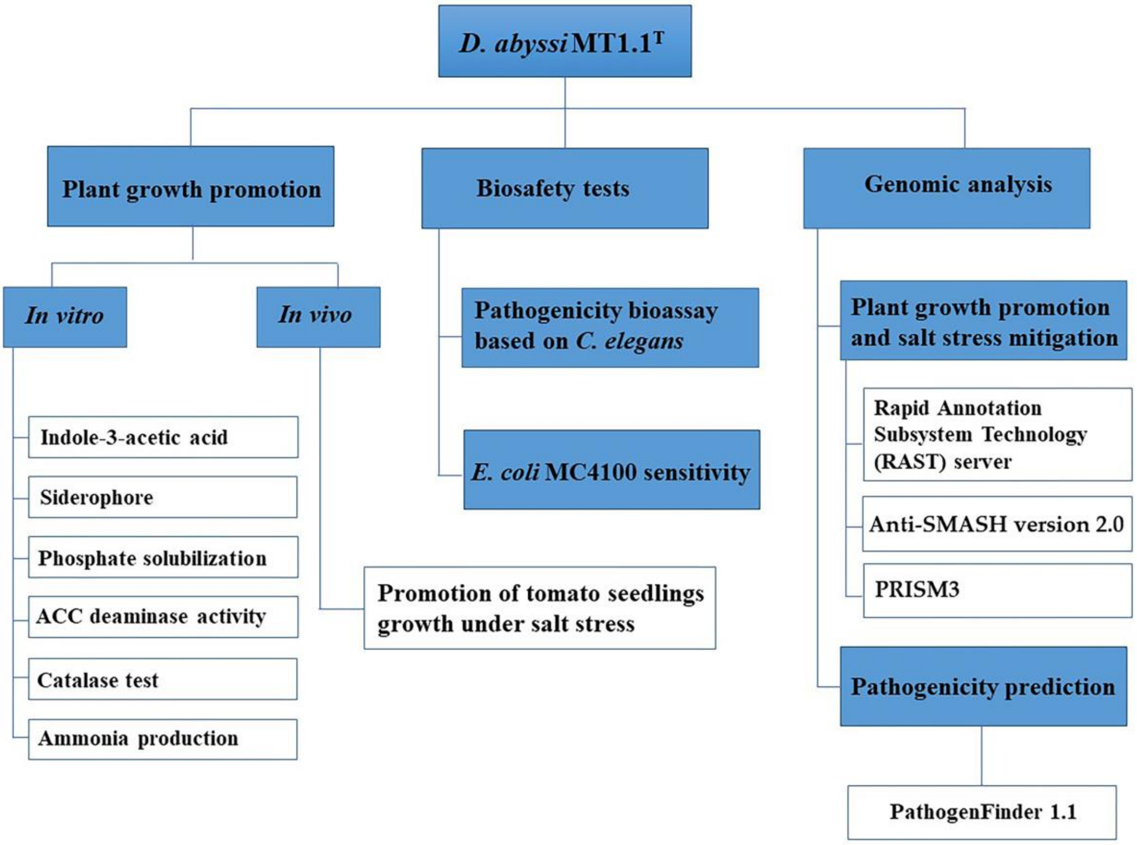

Figure S1. Overviews of all experimental methods used in this study.

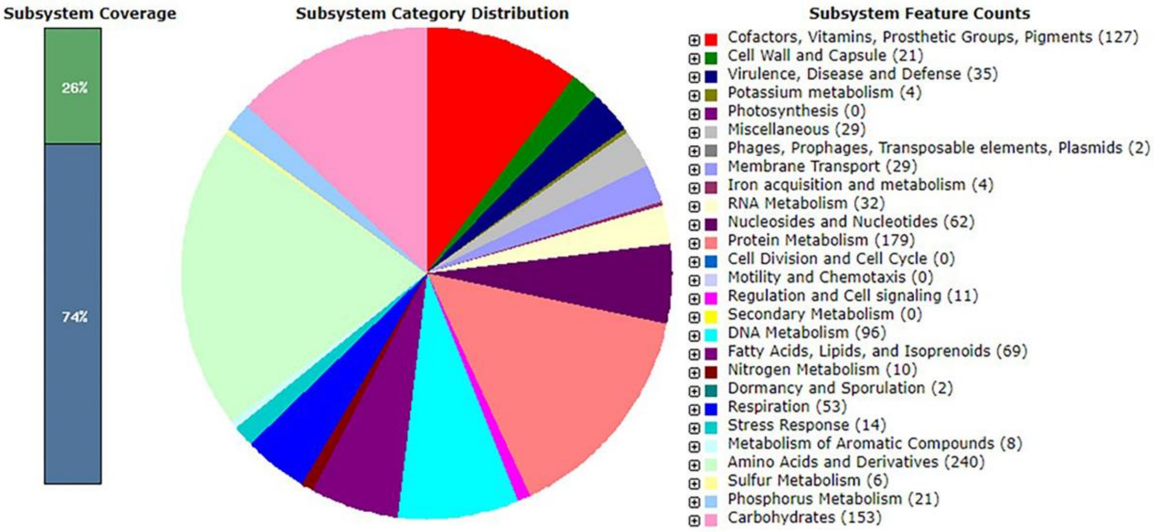

Figure S2. Subsystems of predicted genes in *D. abyssi* MT1.1ᵀ genome using RAST server.
